# MFSD7C switches mitochondrial ATP synthesis to thermogenesis in response to heme

**DOI:** 10.1038/s41467-020-18607-1

**Published:** 2020-09-24

**Authors:** Yingzhong Li, Nikola A. Ivica, Ting Dong, Dimitrios P. Papageorgiou, Yanpu He, Douglas R. Brown, Marianna Kleyman, Guangan Hu, Walter W. Chen, Lucas B. Sullivan, Amanda Del Rosario, Paula T. Hammond, Matthew G. Vander Heiden, Jianzhu Chen

**Affiliations:** 1 Koch Institute for Integrative Cancer Research and Department of Biology, Massachusetts Institute of Technology, Cambridge, MA 02139 USA; 2 Department of Materials Science and Engineering, Massachusetts Institute of Technology, Cambridge, MA 02139 USA; 3 Department of Chemical Engineering, Massachusetts Institute of Technology, Cambridge, MA 02139 USA; 4 Whitehead Institute for Biomedical Research, Cambridge, MA 02142 USA; 5 Dana-Farber Cancer Institute, Boston, MA 02115 USA; 6 Present Address: Boston Combined Residency Program, Department of Pediatrics, Boston Children’s Hospital, Boston, MA 02115 USA

**Keywords:** Mitochondria, Energy metabolism, Signal transduction

## Abstract

ATP synthesis and thermogenesis are two critical outputs of mitochondrial respiration. How these outputs are regulated to balance the cellular requirement for energy and heat is largely unknown. Here we show that major facilitator superfamily domain containing 7C (MFSD7C) uncouples mitochondrial respiration to switch ATP synthesis to thermogenesis in response to heme. When heme levels are low, MSFD7C promotes ATP synthesis by interacting with components of the electron transport chain (ETC) complexes III, IV, and V, and destabilizing sarcoendoplasmic reticulum Ca^2+^-ATPase 2b (SERCA2b). Upon heme binding to the N-terminal domain, MFSD7C dissociates from ETC components and SERCA2b, resulting in SERCA2b stabilization and thermogenesis. The heme-regulated switch between ATP synthesis and thermogenesis enables cells to match outputs of mitochondrial respiration to their metabolic state and nutrient supply, and represents a cell intrinsic mechanism to regulate mitochondrial energy metabolism.

## Abstract (web summary)

Mitochondria maintain a balance between thermogenesis and ATP synthesis, but how this is coordinated is largely unknown. Here, the authors show that MFSD7C coordinates ATP synthesis and thermogenesis in response to heme by directly binding to electron transport chain complexes and SERCA2b.

## Introduction

Energy released from oxidation of carbohydrates and lipids generates a proton gradient across the mitochondrial inner membrane that can be used for ATP synthesis (coupled mitochondrial respiration) and thermogenesis (uncoupled mitochondrial respiration)^1^. While most attention has been focused on the role of mitochondrial respiration in ATP production, it is estimated that in endotherms the majority of the proton-motive force is used for heat generation to maintain a stable body temperature^2,3^. Studies have identified uncoupling protein UCP1 in cellular thermogenesis by transporting protons from the intermembrane space into the matrix of mitochondria^4,5^. In particular, UCP1 is required for heat production by adipocytes of brown adipose tissue^6^, where it is highly expressed. Apart from UCP1, sarcoendoplasmic reticulum Ca^2+^-ATPase 1 (SERCA1) isoform, which hydrolyzes ATP to pump Ca^2+^ from cytosol into the endoplasmic reticulum (ER)^7^, is known to promote thermogenesis in thermogenic organs of certain species of fish^8,9^. Recently, SERCA2b was shown to be required for thermogenesis in beige adipocytes of *Ucp1*^*−/−*^ mice and in pigs^10^, which lack a functional copy of *Ucp1*, while SERCA1 may stimulate thermogenic activity in white adipocytes in mice^11^. Despite these findings, molecular mechanisms that govern whether the energy stored in the mitochondrial proton gradient is used for ATP synthesis or thermogenesis to meet dynamic cellular requirements are largely unknown.

Heme, an iron-containing cyclic tetrapyrrole, belongs to an ancient class of co-factors that support diverse cellular processes. Heme is a co-factor for proteins involved in O_2_ and CO_2_ transport, mitochondrial respiration, redox reactions, circadian rhythm, transcription and translation^12,13^. Directly relevant to energy metabolism, heme is a co-factor for several electron transport chain (ETC) components, where it mediates electron transfer reactions that are coupled to formation of the mitochondrial proton gradient^14^. These observations highlight the critical function of heme in energy metabolism, but whether it plays any role in regulating mitochondrial respiration has not been examined.

Major facilitator superfamily domain containing 7C (MFSD7C), also known as feline leukemia virus subgroup C receptor-related protein 2 (FLVCR2) and solute carrier family 49 member 2 (SLC49A2), is a member of the 12-transmembrane solute carrier family, implicated in proliferative vasculopathy and hydranencephaly-hydrocephaly or Fowler syndrome^15–17^. Truncation and missense mutations in *Mfsd7c* are associated with this autosomal recessive prenatal lethal disorder characterized by multi-organ defects involving brain, kidney and muscle^15,18^. MFSD7C was reported to be a heme transporter based on its binding to heme-conjugated agarose beads and the increased heme uptake by MFSD7C-transfected cells^19^, however a direct role in heme transport has been questioned^20^. To date, the cellular function of MFSD7C and the mechanism by which its mutations cause Fowler syndrome are unknown.

Here we show: (i) MFSD7C resides in the mitochondria and interacts with components of ETC complexes and SERCA2b. (ii) Knockout of *Mfsd7c* results in uncoupled mitochondrial respiration, characterized by increased oxygen consumption rate (OCR) and thermogenesis, a phenotype that is phenocopied by treating parental cells with heme. (iii) The knockout phenotype is corrected by expression of both a full-length and an N-terminal domain (NTD)-truncated MFSD7C, but only the former corrects response to heme. (iv) Mechanistically, binding of heme to the NTD dissociates MFSD7C from ETC components and SERCA2b, leading to SERCA2b stabilization and increased thermogenesis. Our study identifies that MFSD7C switches ATP synthesis to thermogenesis in response to heme, therefore linking the outputs of mitochondrial respiration to the cell’s metabolic state and nutrient supply.

## Results

### MFSD7C binds heme through the N-terminal domain

**Fig. 1:**
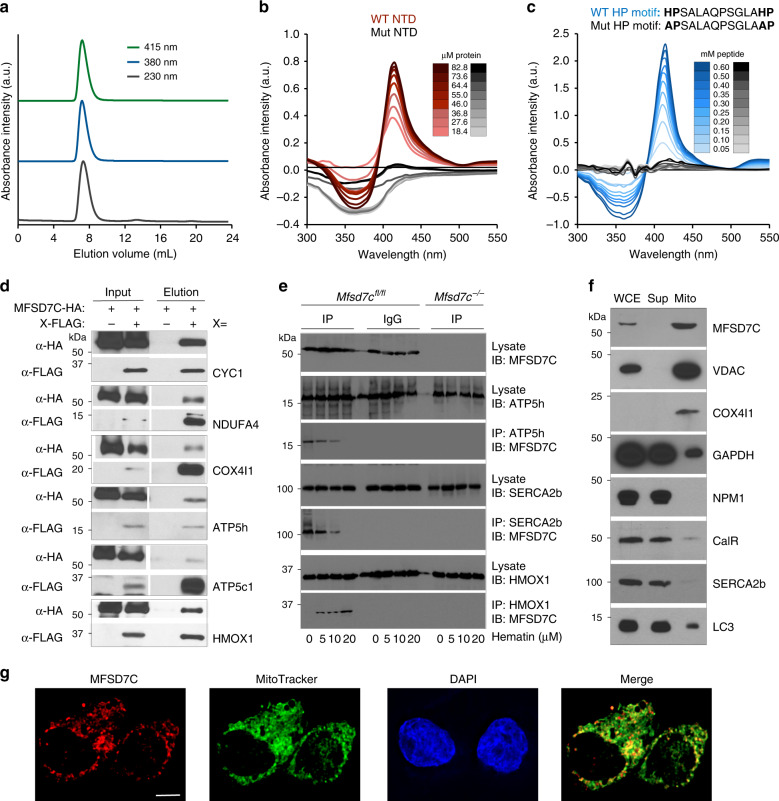
MFSD7C interacts with heme and ETC components in the mitochondria. **a** Superdex 75 gel filtration chromatograms of human NTD and heme. NTD was incubated with heme and run on Superdex 75 gel filtration column. The flow through was measured for absorbance at 230 nm (gray), 380 nm (blue), and 415 nm (green). Absorbance intensity was normalized to maximum value. **b** Changes in absorption spectrum intensity of heme incubated with different concentrations of wild-type (red) or mutant (gray) NTD (see color scale). Heme (100 µM) absorption was set to zero. **c** Changes in absorption spectrum intensity of heme (100 µM) incubated with different concentrations of wildtype (blue) or mutant (gray) HP motif peptide (see color scale). Wildtype (WT) and mutant (Mut) peptide sequences are shown. **d** Co-IP and immunoblotting analysis of HA-tagged MFSD7C and FLAG-tagged CYC1, NDUFA4, COX4I1, ATP5h, ATP5c1, or HMOX1. Shown are representative data from five separate experiments. **e** Co-IP and immunoblotting analysis of endogenous MFSD7C with ATP5h, SERCA2b and HMOX1 in bone marrow-derived macrophages from *Mfsd7c*^*fl/fl*^ or *Mfsd7c*^*−/−*^ C57BL/6 mice (see “Methods” section for details). Shown are immunoblots of MFSD7C, ATP5h, SERCA2b, and HMOX1 on whole lysates or immunoblots of MFSD7C on anti-ATP5h, anti-SERCA2b and anti-HMOX1 immunoprecipitates. Representative data from one from three experiments are shown. **f** Mouse whole brain extract was fractionated using differential centrifugation to enrich for mitochondria and analyzed by immunoblotting against the indicated proteins. Shown are representative data from three separate experiments. WCE: whole cell extract, Sup: supernatant, Mito: 10,000×*g* mitochondrial fraction. **g** Immunofluorescent localization of MFSD7C in mitochondria. THP-1 cells were stained with MitoTracker (green), fixed and permeabilized, and then stained with rabbit polyclonal antibody specific for the C-terminus of MFSD7C, followed with Alexa Fluor® 594-labeled goat anti-rabbit antibody (red). Nuclei were labeled using DAPI (blue). Co-localization between MFSD7C and MitoTracker appears as yellow in the merged images. Scale bar in **d** and **f**: 10 µm.

Sequence and structural analyses predict that MFSD7C belongs to the 12-transmembrane solute carrier family. The NTD of human and mouse MFSD7C contains five regularly spaced histidine-proline (HP) repeats, a feature conserved in many mammalian species (Supplementary Fig. 1a). Histidine is an axial ligand to the central heme-iron in many heme-binding proteins^21^ and MFSD7C was reported to precipitate with hemin agarose^19^. To test whether the NTD directly binds to heme, the 84 amino acid NTD of human MFSD7C was recombinantly expressed and purified (Supplementary Fig. 1b). In a gel-filtration chromatography, heme (616 Da) co-eluted with the NTD (8.6 kDa) as indicated by heme-specific absorbance at 380 nm and 415 nm and NTD-specific absorbance at 230 nm of the protein-containing fractions (Fig. 1a and Supplementary Fig. 1c, d). When heme was incubated with the NTD, a concentration-dependent increase in the intensities of the Soret band (415 nm) and the Q band (535 nm) was detected (Fig. 1b), and the rate of increase of the Soret band intensities with respect to NTD concentration suggests two or more heme binding sites per NTD (Supplementary Fig. 1e). The absorbance shift was abolished when all five histidine residues in the HP repeats were mutated to alanine (Fig. 1b). The isothermal titration calorimetry analysis showed that the NTD binds three heme molecules with two strong binding sites (K_D_ ~ 1 µM) and one weaker site (K_D_ ~ 220 µM) (Supplementary Fig. 1f). Similarly, when incubated with a synthetic 14-amino acid HP motif peptide, heme absorption spectrum also showed a concentration-dependent increase in Soret band and Q band peaks at a rate consistent with one heme bound to one HP motif peptide at a K_D_ of ~1 µM (Fig. 1c and Supplementary Fig. 1e, g). The absorbance shift was abolished when both histidine residues in the HP motif peptide were mutated to alanine (Fig. 1c). These results show that the NTD of human MFSD7C is capable of binding to 2–3 heme molecules.

### MFSD7C interacts with components of ETC complex III, IV, and V

We used immunoprecipitation (IP) and mass spectrometry (MS) to identify proteins that interact with MFSD7C. Because an anti-MFSD7C monoclonal antibody is not available, we used MFSD7C tagged with both Myc and FLAG epitopes in a MFSD7C-expressing murine alveolar macrophage cell line MH-S (Supplementary Fig. 2a–c and see “Methods” section and PRIDE (http://www.ebi.ac.uk/pride/archive/projects/PXD021016 for details). A total of 58 proteins were identified excluding MFSD7C and common contaminants of IP experiments such as keratin, actin, and ribosomal proteins (Table 1). Twenty-six of the 58 proteins are annotated as mitochondrial proteins and 4 (three Fc receptors and one integrin) are known to reside in the cytoplasmic membrane. The 58 proteins were classified into 10 different functional categories based on gene ontology.

**Table 1:** List of proteins co-immunoprecipitated with MFSD7C.

Classification	Protein	Localization
Heme metabolism	MFSD7C, HMOX1, Tfrc	N/A
Mitochondrial respiration	CYC1, NDUFA4, COX4I1, ATP5h, ATP5c1	Mitochondria
Adenine nucleotide translocase	Slc25a4, Slc25a5	Mitochondria
Calcium signaling	Atp2a2 (SERCA2b), Canx, Ssr4, Hax1, Slc3a2	N/A
Metabolism	Acsl5, Elovl1, Mthfd1l, Cds2, Asph	Mitochondria
Other mitochondrial proteins	Dnaja1, Immt, TIM50, Afg3l2, Phb, Dnaja3, Tmx3, Rdh13, Sqrdl, Tspo	Mitochondria
Fc receptor and Integrin	Fcer1g, Fcgr3, Fcgr1, Itgb2	Plasma membrane
Other solute carriers	Slc25a3, Slc33a1, Slc25a22, Slc25a11 ***Slc16a1***, ***Slc43a2***	Mitochondria ***N/A***
Posttranslational modification	G3bp2, Oas1a, Rnf126, Amfr, Cpt1a, Cap1, STT3B, Mlec, Atxn10, Tm9sf3, Ptpn6, Lpxn, Nufip2, C2orf18	N/A
Trafficking	Tmem165, Unc93b, Emd, Scamp3, Sec22b	N/A

To validate the IP-MS results, we tested interactions between MFSD7C and heme oxygenase-1 (HMOX1) and all five ETC proteins: CYC1 (complex III), NDUFA4 and COX4I1 (complex IV), ATP5h and ATP5c1 (complex V/ATP synthase) by co-IP. Plasmids encoding HA-tagged MFSD7C and FLAG-tagged candidate proteins were co-transfected into HEK 293T cells and cell lysates were precipitated with anti-FLAG and anti-HA antibodies sequentially, followed by Western blotting with anti-HA or anti-FLAG antibodies. MFSD7C co-precipitated with all six proteins tested (Fig. 1d). To determine whether the observed interactions occur between endogenous proteins, we performed co-IP between MFSD7C and ATP5h, SERCA2b (ATP2a2) and HMOX1 using cell lysates of bone marrow-derived macrophages from C57BL/6 mice with exon 2 of *Mfsd7c* floxed (*Mfsd7c*^*fl/fl*^) or deleted (*Mfsd7c*^*−/−*^) specifically in macrophages (see “Methods” section for details). The endogenous MFSD7C co-immunoprecipitated with endogenous ATP5h, SERCA2b and HMOX1 in *Mfsd7c*^*fl/fl*^ macrophages but not in *Mfsd7c*^*−/−*^ macrophages (Fig. 1e). Thus, MFSD7C likely interacts with mitochondrial ETC components and ER proteins SERCA2b and HMOX1.

To determine MFSD7C subcellular localization, we performed subcellular fractionation followed by Western blotting on whole mouse brain where MFSD7C is strongly expressed^22^. MFSD7C was only detectable in the mitochondrial fraction (10,000×*g* precipitate), along with mitochondrial markers VDAC and COX4I1 (Fig. 1f). In contrast, known cytoplasmic proteins GAPDH and nucleophosmin 1 (NPM1), ER protein calreticulin (CalR) and SERCA2b, and autophagosomal protein LC3 were predominantly present in the supernatant fraction. Furthermore, staining of human monocytic THP-1 cells with a polyclonal antibody specific for the C-terminus of MFSD7C, MitoTracker, and DAPI showed co-localization of MFSD7C and MitoTracker signals (Fig. 1g), with a Pearson’s correlation coefficient value of 0.6. In contrast, the polyclonal antibodies did not stain *Mfsd7c* knockout cells (Supplementary Fig. 2d), suggesting their specificity to MFSD7C. Similarly, when MFSD7C-GFP fusion protein was expressed in HEK 293T cells, most of the GFP signal co-localized with MitoTracker (Supplementary Fig. 2e).

Together, these data suggest that MFSD7C primarily resides in mitochondria where it interacts with components of the ETC complexes III, IV and V.

### MFSD7C regulates coupling of mitochondrial respiration

**Fig. 2:**
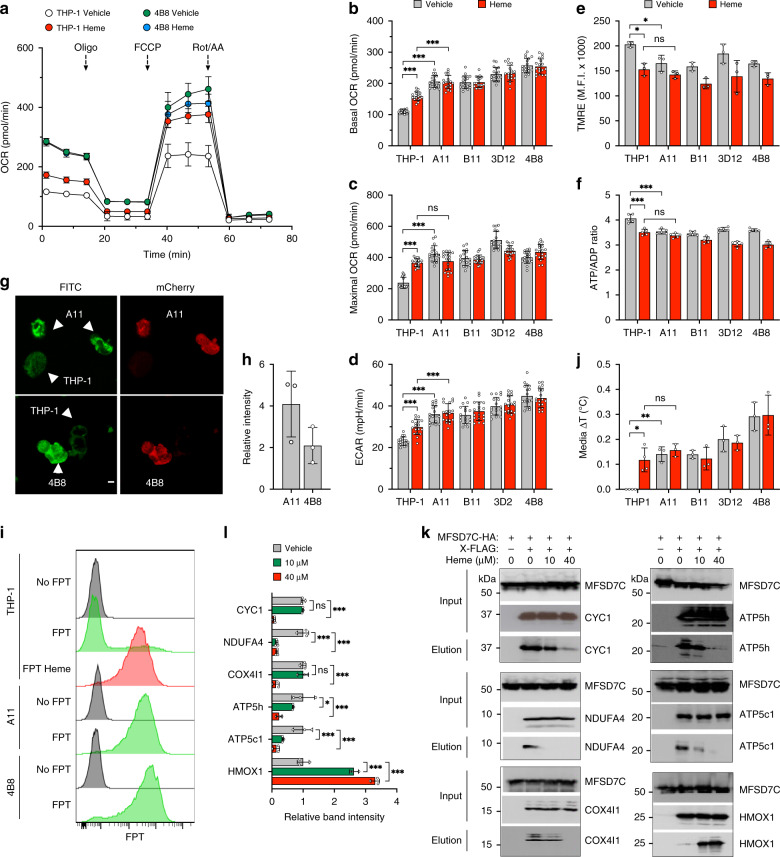
MFSD7C and heme regulate coupling of mitochondrial respiration. **a–f** Comparison of mitochondrial respiratory activities between parental THP-1 cells and four *Mfsd7c* knockout clones (A11, B11, 3D12, and 4B8). Parental and knockout THP-1 cells were cultured in the presence of either vehicle or 40 µM heme for 1 h. **a** Representative OCR measurements of THP-1 cells and 4B8 knockout clone under the indicated conditions. Comparison of basal OCR (**b**), maximal OCR (**c**), and ECAR (**d**), between THP-1 cells and 7CKO clones from three separate experiments. Each dot represents a technical replicate (**b**–**d**, *n* = 18 independent experiments). **e** MMP was measured using TMRE (200 nM) by flow cytometry (*n* = 3 independent experiments). **f** Cellular ATP/ADP ratio (*n* = 5 independent experiments). **g**, **h** Comparison of thermogenesis between parental THP-1 cells and two 7CKO clones (A11 and 4B8) by microscopy using FPT. Green channel detects the FPT intensity and the red channel distinguishes knockout cells, which expressed mCherry, from the parental THP-1 cells. Shown are representative images (**g**) and FPT intensity of A11 and 4B8 relative to THP-1 cells from three separate experiments (**h**). **i** Comparison of thermogenesis between THP-1 and 7CKO cells by flow cytometry. FPT fluorescence intensity was quantified by flow cytometry. Shown are representative plots from three separate experiments. **j** Comparison of temperature changes in THP1 (*n* = 4 independent samples) and 7CKO (*n* = 3 independent samples) culture media (ΔT) with or without heme treatment as measured by thermocouples. **k** and **l** Cells were not treated or treated with heme for 1 h before lysis. Cell lysates were precipitated with anti-FLAG antibody, eluted with FLAG peptide, then precipitated with anti-HA antibody, and eluted with HA peptide, and finally subjected to immunoblotting with anti-HA and anti-FLAG antibodies. Input is the total cell lysate. Shown are representative co-IP/immunoblots (**k**) and average band intensities with standard deviation from three independent experiments (**l**). *p* values were calculated using two-way ANOVA (*p < 0.05, **p < 0.01, ***p < 0.005). Data are presented as mean value ± standard deviation.

To investigate the function of MFSD7C, we generated four independent *Mfsd7c* knockout clones (A11, B11, 3D12 and 4B8, collectively referred to as 7CKO cells) using CRISPR-Cas9 genome editing in THP-1 cells, which had readily detectable levels of MFSD7C but not UCP1 (Supplementary Fig. 3). We assayed oxygen consumption rate (OCR), extracellular acidification rate (ECAR), mitochondrial membrane potential (MMP), and energy charge (ATP/ADP ratio) in the parental THP-1 and 7CKO cells in the absence or presence of heme. Both the basal and maximal OCR were 1.5-2-fold higher in 7CKO cells than in the parental THP-1 cells (Fig. 2a–c and Supplementary Fig. 4a). Heme treatment increased both the basal and maximal OCR of the parental THP-1 cells but not the 7CKO cells. ECAR was significantly higher in 7CKO cells than in the parental THP-1 cells (Fig. 2d). In contrast, MMP and ATP/ADP ratio were ~10–25% lower in 7CKO cells than in THP-1 cells and heme treatment reduced MMP and ATP/ADP ratio of THP-1 cells to the similar levels as in 7CKO cells (Fig. 2e, f). By targeted metabolomic analysis, the relative amounts of ATP, ADP, and AMP were different between THP-1 and 7CKO clone B11, and the ATP/ADP ratio was significantly reduced in B11 compared to parental THP-1 cells (Supplementary Fig. 4b, c). These results show that heme and *Mfsd7c* knockout have similar effects on OCR, MMP and cellular energy charge. The observation of increased OCR and ECAR without increase of MMP or energy charge is consistent with inefficient ATP production resulting from uncoupled mitochondrial respiration.

We measured the effect of *Mfsd7c* knockout and heme on thermogenesis using a temperature-sensitive dye: fluorescent polymeric thermometer (FPT, see “Methods” section and Supplementary Fig. 5a, b for details). As previously reported^23^, FPT fluorescent intensities increased when cells were incubated at increasing temperatures (Supplementary Fig. 5c). To compare thermogenesis between the parental THP-1 cells and 7CKO cells under the same condition, we co-cultured THP-1 cells with either A11 or 4B8 7CKO cells, loaded the cells with FPT, and imaged FPT intensity in the same culture well. The FPT fluorescent intensity was visibly higher in mCherry-positive A11 and 4B8 cells than the parental THP-1 cells in the same microscopy field (Fig. 2g). Quantification of the fluorescent intensity showed a 2-4-fold increase in 7CKO cells compared to THP-1 cells (Fig. 2h). Consistently, only a small fraction of THP-1 cells had an increased FPT fluorescence by flow cytometry, whereas almost all cells had dramatically increased fluorescence following heme treatment for 1 h (Fig. 2i). In contrast, 7CKO clones A11 and 4B8 had similarly high FPT fluorescence without heme treatment. As a complementary approach, we measured the temperature of culture media using thermal couples (See “Methods” section for details). The temperature of the 7CKO culture was 0.15–0.3 °C higher than the THP-1 culture (Fig. 2j). Heme treatment increased the temperature of THP-1 culture by 0.12 °C but not 7CKO cell cultures.

To validate the observed effects in different cell types, we knocked out *Mfsd7c* in human breast cancer MCF7 cells and human embryonic kidney 293T cells using CRISPR-Cas9 genome editing (Supplementary Fig. 6a). Compared to the parental MCF7 and HEK293T cells, *Mfsd7c* knockout cells exhibited significantly higher OCR and FTP intensity (Supplementary Fig. 6b–f). Similarly, heme treatment of parental cells significantly stimulated OCR and FTP intensity. Collectively, these data show that both *Mfsd7c* knockout and heme treatment of THP-1, MCF7, and HEK293T cells promote uncoupled mitochondrial respiration.

**Fig. 3:**
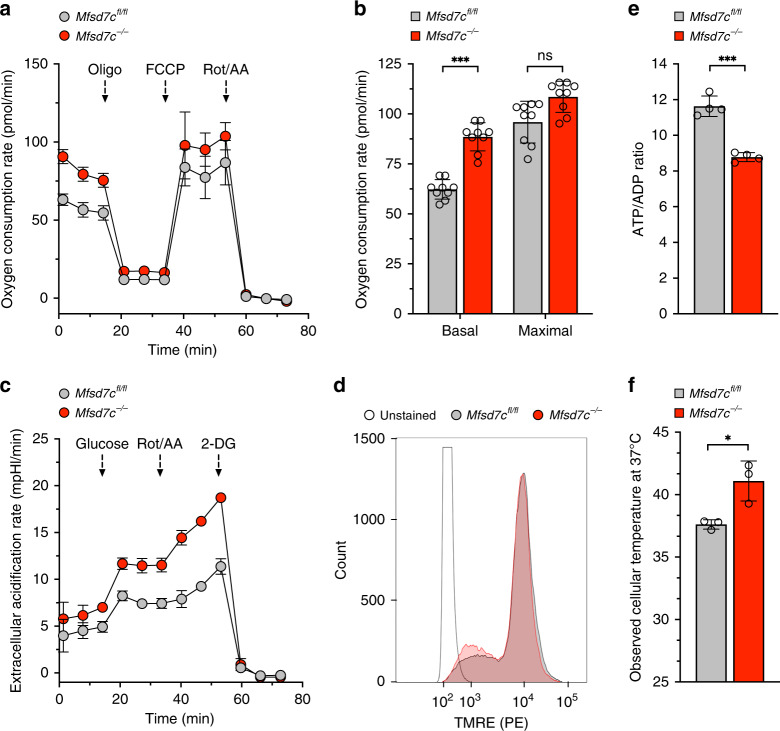
Loss of *Mfsd7c* stimulates OCR, ECAR, and thermogenesis in BMDM. Bone marrow-derived macrophages (BMDM) were derived from exon 2 floxed (*Mfsd7c*^*fl/fl*^) C57BL/6 mice and C57BL/6 mice with exon 2 deletion in macrophages (*Mfsd7c*^*−/−*^). **a**, **b** Representative OCR analysis from *Mfsd7c*^*fl/fl*^ and *Mfsd7c*^*−/−*^ macrophages as measured by Seahorse XF96e Analyzer and their responses to oligomycin (oligo), FCCP, and rotenone plus antimycin A (Rot/AA) (*n* = 3 technical replicates) (**a**), and their average basal and maximal OCR (**b**) (*n* = 9 independent experiments from 3 biological replicates). **c** Representative ECAR analysis of *Mfsd7c*^*fl/fl*^ and *Mfsd7c*^*−/−*^ macrophages and their responses to glucose, Rot/AA, and 2-deoxyglucose (2-DG) (*n* = 3 technical replicates). **d** Mitochondrial membrane potential of *Mfsd7c*^*fl/fl*^ and *Mfsd7c*^*−/−*^ macrophages analyzed with TMRE staining followed by flow cytometry (1 representative histogram picked from 3 biological replicates). **e** Cellular ATP/ADP ratio, (*n* = 4 biological replicates from average of 5 technical replicates). **f** Estimates of cellular temperature of *Mfsd7c*^*fl/fl*^ and *Mfsd7c*^*−/−*^ macrophages as measured by fluorescent thermoprobe (*n* = 3 biological replicates). *p* values were calculated using unpaired *t*-test (*p < 0.05, **p < 0.01, ***p < 0.005). Data are presented as mean value ± standard deviation.

To test whether loss of *Mfsd7c* uncouples mitochondrial respiration in primary cells, we created a C57BL/6 mouse strain with *loxP* sites flanking exon 2 of *Mfsd7c (Mfsd7c*^*fl/fl*^*)*. *Mfsd7c*^*lf/fl*^ mice were crossed with *LysMcre* mice to deplete *Mfsd7c* (*Mfsd7c*^*−/−*)^ specifically in myeloid cells (Supplementary Fig. 7a–c). We generated bone marrow-derived macrophages (BMDM) by culturing bone marrow cells from *Mfsd7c*^*fl/fl*^ and *Mfsd7c*^*−/−*^ mice for 7 days (see “Methods” section for details). BMDM expressed the macrophage markers F4/80 and CD11b (Supplementary Fig. 7d), and were confirmed for deletion of *Mfsd7c* locus and near complete loss of both *Mfsd7c* transcript and protein (Supplementary Fig. 7e–g). Compared to *Mfsd7c*^*fl/fl*^ BMDM, *Mfsd7c*^*−/−*^ BMDM had significantly higher levels of OCR and ECAR (Fig. 3a–c). Although MMP was not significantly different, ATP/ADP ratio was significantly decreased in *Mfsd7c*^*−/−*^ BMDM (Fig. 3d, e). We measured thermogenesis of *Mfsd7c*^*fl/fl*^ and *Mfsd7c*^*−/−*^ BMDM using a commercial cellular thermoprobe dye (Funakoshi). The uptake of the thermoprobe polymer was similar between *Mfsd7c*^*−/−*^ and *Mfsd7c*^*fl/fl*^ BMDM (Supplementary Fig. 8a–c), but the temperature sensitive fluorescence was significantly higher at several different incubation temperatures (Supplementary Fig. 8d–f). We estimated that *Mfsd7c*^*−/−*^ BMDM were on average 4^o^C hotter than *Mfsd7c*^*fl/fl*^ BMDM (Fig. 3f). These results show that loss of *Mfsd7c* in macrophages results in increased thermogenesis, as was the case with THP-1 7CKO cell lines.

The fact that heme treatment phenocopies the effect of *Mfsd7c* knockout suggests that heme may work by disrupting MFSD7C interactions with ETC components. To test this hypothesis, we performed co-IP between MFSD7C and the ETC components with or without treating the cells with 10 μM or 40 μM heme for 1 h before cell lysis. As expected, CYC1, NDUFA4, COX4i1, ATP5h, and ATP5c1 co-precipitated with MFSD7C without heme treatment (Fig. 2k, l). With increasing level of heme treatment, less or very little ETC components co-precipitated with MFSD7C. In contrast, interaction between HMOX1 and MFSD7C was enhanced by heme treatment. Similarly, heme also disrupted interactions between endogenous MFSD7C and ATP5h but enhanced interactions between endogenous MFSD7C and HMOX1 in bone marrow-derived macrophages (Fig. 1e). These results suggest that MFSD7C normally inhibits OCR and thermogenesis by interacting with ETC components and that heme stimulates OCR and thermogenesis by disrupting MFSD7C interactions with ETC components, thus phenocopying the effects of *Mfsd7c* knockout.

### The N-terminal domain of MFSD7C mediates response to heme

**Fig. 4:**
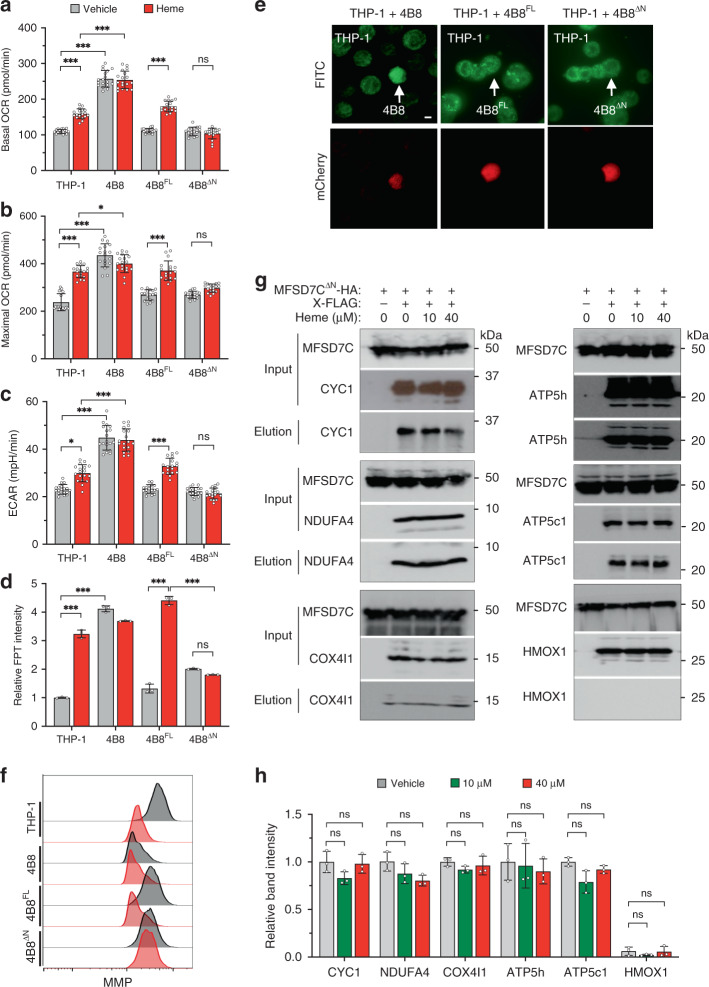
NTD of MFSD7C mediates heme effect on mitochondrial respiration. **a–c** Comparison of basal OCR (**a**), maximal OCR (**b**), and ECAR (**c**) of THP-1, 4B8, and 4B8^FL^, and 4B8^ΔN^ cells treated with vehicle or heme for 1 h (*n* = 18 separate experiments). **d** Comparison of FPT intensity of THP-1, 4B8, 4B8^FL^, and 4B8^ΔN^ cells. Cells were incubated with FPT for 6 h, washed and reseeded in poly-lysine coated glass bottom dishes. After cells attached to the glass, medium containing 40 μM heme was added. Cells in the same field were imaged immediately and again 1 h later. Relative FPT fluorescent intensities, normalized to THP-1, from three experiments are shown. Representative images are shown in Supplementary Fig. 9e. (*n* = 3 independent experiments). **e** Restoration of thermogenesis in 7CKO cells by expression of MFSD7C^FL^ and MFSD7C^ΔN^. THP-1 cells were co-cultured with 4B8, 4B8^FL^, or 4B8^ΔN^ in FPT solution for 6 h, washed and reseeded in poly-lysine coated glass bottom dishes. Cells were imaged by confocal laser-scanning microscopy. Green channel shows the FPT intensity and the red channel (mCherry) distinguishes knockout cells from the parental THP-1 cells. Representative images from three experiments are shown. Scale bar: 10 µm. **f** Heme treatment reduces MMP in 4B8 cells complemented with MFSD7C^FL^ but not MFSD7C^ΔN^. THP-1, 4B8, 4B8^FL^, and 4B8^ΔN^ cells were treated with vehicle or heme for 1 h and MMP was measured by using JC-10 Mitochondrial Membrane Potential Assay Kit followed by flow cytometry. Representative mitochondrial staining profiles from three experiments are shown. **g** and **h** Heme does not disrupt MFSD7C^ΔN^ interactions with ETC components. Co-IP was performed as in Fig. 2k, except HA-tagged MFSD7C^ΔN^ was used. Shown are representative Western blotting data (**g**) and quantification of band intensities from *n* = 3 independent experiments (**h**). *p* values were calculated using two-way ANOVA (*p < 0.05, **p < 0.01, ***p < 0.005). Data are presented as mean value ± standard deviation.

To delineate the relationship between the heme-binding by the NTD in vitro and the effect of heme on OCR and thermogenesis in vivo, we complemented 7CKO clone 4B8 with either the full length MFSD7C (MFSD7C^FL^) or MFSD7C lacking the first 80 amino acid residues of the NTD (MFSD7C^ΔN^) to generate 4B8^FL^ and 4B8^ΔN^ cells, respectively (Supplementary Fig. 9a). Expression of the full length and truncated MFSD7C were confirmed, as well as their localization to mitochondria (Supplementary Fig. 9b, c). Expression of MFSD7C^FL^ or MFSD7C^ΔN^ fully rescued *Mfsd7c* knockout phenotype as evident from decreased OCR, ECAR and FPT intensity, and increased MMP (Fig. 4a–f and Supplementary Fig. 9d, e). Heme treatment of 4B8^FL^ cells phenocopied the parental THP-1 response to heme, including increased OCR, ECAR, FPT intensity but decreased MMP. In contrast, 4B8^ΔN^ cells showed little to no response to heme. Furthermore, heme failed to disrupt the interactions between MFSD7C^ΔN^ and CYC1, NDUFA4, COX4i1, ATP5h, and ATP5c1 (Fig. 4g, h). Notably, MFSD7C^ΔN^ did not co-precipitate with HMOX1 either with or without heme treatment, suggesting that the NTD is required for MFSD7C interaction with HMOX1. These results show that heme regulates mitochondrial respiration specifically through the NTD of MFSD7C in vivo, consistent with direct binding of the NTD to heme in vitro. The NTD is not required for MFSD7C interactions with ETC components, providing a molecular explanation for the restoration of mitochondrial respiration by MFSD7C^ΔN^ without restoring the response to heme.

### MFSD7C regulates thermogenesis by degradation of SERCA2b

**Fig. 5:**
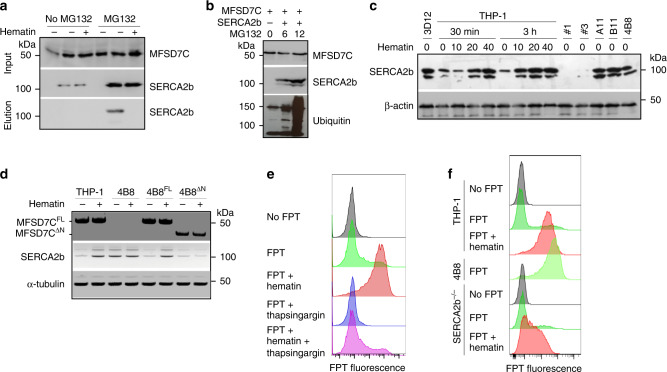
MFSD7C and heme regulate thermogenesis through SERCA2b in THP-1 cells. **a** 293FT cells were transfected with HA-tagged murine MFSD7C and FLAG-tagged murine SERCA2b. Thirty hours later, MG132 was added into half of the cells and the other half was not treated. Another 35 h later, some cells were treated with 40 μM heme for 1 h before lysis. Cell lysates were immunoprecipitated with anti-FLAG antibody, and eluted, followed by anti-HA immunoprecipitation and elution. Total cell lysate and elute were subjected to Western blotting and probed with anti-SERCA2b or anti-MFSD7C antibodies. Shown are representative data from one of three experiments. **b** 293T cells were co-transfected with HA-MFSD7C and FLAG-SERCA2b. Twenty-four hours later, cells were either not treated or treated with 10 μM MG132 for either 6 or 12 h. The cells were lysed and subjected to FLAG pull-down and blotted with anti-MFSD7C, anti-SERCA2b and anti-ubiquitin antibodies. Shown are representative data from one of three experiments. **c** SERCA2b protein levels in parental THP-1 cells, 7CKO cells (3D12, A11, B11, and 4B8) or *SERCA2b*^*−/−*^ (#1 and #3) cells. Parental THP-1 cells were either not treated or treated with heme before lysis. Shown are representative data from one of four experiments. **d** THP-1, 4B8, 4B8^FL^, and 4B8^ΔN^ cells were either not treated or treated with 40 μM heme for 1 h, lysed and subjected to Western blotting with anti-MFSD7C (top), anti-SERCA2b (middle), and anti-®-tubulin (bottom). Shown are representative data from one of two experiments. **e** THP-1 cells were incubated with FPT for 6 h, washed, treated with or without 4 μM thapsigargin for 2 h. Cells were washed and treated with 40 μM heme for 1 h before flow cytometry. Representative FPT histograms from one of three experiments are shown. **f** Parental and *Serca2b*^*−/−*^ THP-1 cells were incubated with FPT for 6 h, washed and treated with or without 40 μM heme for 1 h, followed by flow cytometry. Shown are representative FPT histograms from one of three experiments.

How does *Mfsd7c* knockout or heme treatment induce thermogenesis? We noticed that SERCA2b (a.k.a. ATP2a2) was identified as a MFSD7C-interacting protein by IP-MS (Table 1). We validated the interaction by co-IP in 293T cells. In the absence of proteasome inhibitor MG132, a low level of SERCA2b was detected but none was co-precipitated with MFSD7C (Fig. 5a). In the presence of MG132, a significantly higher level of SERCA2b was detected, and an appreciable level was co-precipitated with MFSD7C. The observed co-IP was abolished when cells were treated with heme for 1 h. Similarly, co-IP of endogenous MFSD7C and SERCA2b was observed in bone marrow-derived wild-type but not *Mfsd7c*^*−/−*^ macrophages, and the co-IP was diminished following heme treatment (Fig. 1e). Consistently, MFSD7C co-localized with SERCA2b with a Pearson’s correlation coefficient value of 0.89, and this value was reduced to 0.67 following heme treatment (Supplementary Fig. 10a–c). A fraction of MFSD7C was also co-localized with the ER marker at the mitochondrial-ER contact junction (Supplementary Fig. 10d, e), a known contact point between the two organelles^24^. These results suggest that MFSD7C interacts with SERCA2b and that this interaction is disrupted by heme.

Stabilization of SERCA2b by proteasome inhibitor MG132 suggests that MFSD7C may promote SERCA2b degradation through ubiquitination and subsequent proteasomal degradation. To test this hypothesis, 293T cells were transfected with FLAG-tagged SERCA2b and treated with MG132 for either 6 or 12 h, lysed, immunoprecipitated with anti-FLAG, and Western blotted with anti-MFSD7C, anti-SERCA2b and anti-ubiquitin antibodies. With longer MG132 treatment, more full-length and ubiquitinated SERCA2b was precipitated (Fig. 5b). Furthermore, SERCA2b level was significantly higher in THP-1 cells following heme treatment and in 7CKO cells than in parental THP-1 cells (Fig. 5c). Expression of both MFSD7C^FL^ and MFSD7C^ΔN^ in 4B8 cells led to a reduction of SERCA2b, which was restored by heme treatment in 4B8^FL^ but not 4B8^ΔN^ cells (Fig. 5d). These results show that interactions between MFSD7C and SERCA2b leads to ubiquitin-mediated degradation of SERCA2b, and heme disrupts the interaction and therefore stabilizes SERCA2b.

To investigate the role of SERCA2b in MFSD7C/heme-regulated thermogenesis, we tested if heme-stimulated thermogenesis is inhibited by thapsigargin, a known SERCA2b inhibitor^25^. Indeed, heme-stimulated thermogenesis in THP-1 cells was mostly inhibited by thapsigargin (Fig. 5e and Supplementary Fig. 10f). Consistently, thermogenesis of *Serca2b*^*−/−*^ THP-1 cells was significantly diminished following heme treatment (Fig. 5f). These results support a critical role for SERCA2b in mediating MFSD7C-regulated and heme-regulated thermogenesis.

## Discussion

**Fig. 6:**
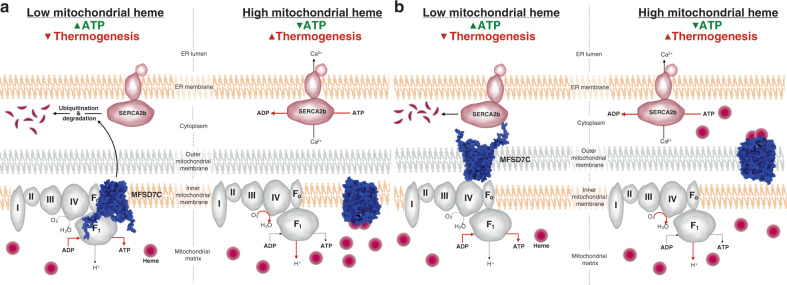
Proposed MFSD7C-regulated cellular thermogenesis model. **a**, **b** Proposed models of MFSD7C regulation of mitochondrial respiration in response to heme, with MFSD7C residing in the inner (**a**) or the outer (**b**) mitochondrial membrane. When heme level is low, MFSD7C interacts with ETC components and SERCA2b, leading to SERCA2b ubiquitination and degradation and coupled mitochondrial respiration: increased ATP synthesis and reduced thermogenesis. When heme level is high, heme binding to the NTD of MFSD7C disrupts its interactions with ETC components and SERCA2b, leading to SERCA2b stabilization and uncoupled mitochondrial respiration: increased thermogenesis and reduced ATP synthesis.

We identify MFSD7C as a heme-regulated switch that controls the coupling of mitochondrial respiration. Our biochemical analyses support MFSD7C interactions with heme, ETC complexes, and SERCA2b. At least for the recombinant NTD, our findings suggest binding of 2–3 heme molecules, consistent with the NTD of human MFSD7C containing two and a half heme-binding HP motifs. The fact that only five proteins from the ETC complexes were co-precipitated with MFSD7C in our IP-MS analysis suggests there is selectivity of MFSD7C interactions with ETC components, consistent with the relatively stringent cell lysis and washing conditions (0.1% NP-40) used in the immunoprecipitation. Our data suggest that MFSD7C interacts with SERCA2b at the mitochondrial-ER contact junction. Importantly, we find that MFSD7C interactions with ETC components and SERCA2b are disrupted by heme and in particular heme stabilizes SERCA2b, which is ubiquitinated and degraded in the presence of MFSD7C. These dynamic interactions shed light on the mechanism by which MFSD7C regulates coupling of mitochondrial respiration in response to heme: when heme levels are low, MFSD7C interacts with ETC components and SERCA2b, leading to SERCA2b degradation and coupled mitochondrial respiration; upon heme binding to the NTD of MFSD7C the interactions are disrupted, leading to SERCA2b stabilization and uncoupling of mitochondrial respiration (Fig. 6a, b). It is also possible that the interactions of MFSD7C with ETC components could promote assembly of supercomplexes and therefore coupled mitochondrial respiration^26^.

Our findings suggest heme is an endogenous metabolite that is sensed by MFSD7C in the regulation of mitochondrial respiration. The observation that the NTD of MFSD7C binds to 2–3 heme molecules could enable MFSD7C to respond to a range of heme concentrations. As a metabolite, heme is well suited as a proxy for monitoring the metabolic state and nutrient supply of the cell. First, heme is a co-factor for several ETC components and directly mediates electron transport reactions so that the mitochondrial heme level likely reflects the ETC capacity^27^. Second, heme biosynthesis starts and finishes in the lumen of mitochondria. The rate limiting first step uses succinyl-CoA and glycine, which are, respectively, intermediates of tricarboxylic acid cycle and one-carbon metabolism, two important outputs of mitochondrial metabolism^28,29^. The level of heme therefore reflects the metabolic state of the cell. Third, heme contains an iron and may reflect iron availability, and because it is absorbed from food, it might also reflect nutritional status at the organismal level. In fact, increased thermogenesis after a meal has been known since ancient times^30^ and food has been classified based on their thermogenic properties^31^. Not surprisingly, meat, especially heme-abundant red meat, is the most thermogenic. However, the molecular basis underlying the thermic effect of food is largely unknown. Our findings suggest a possible new mechanism: heme absorbed from food stimulates thermogenesis by uncoupling mitochondrial respiration. Thus, the MFSD7C-mediated switch between ATP synthesis and thermogenesis in response to heme links the outputs of mitochondrial respiration to the cell’s metabolic state and nutrient supply.

Our findings shed light on how dysfunctional mutations in *Mfsd7c* may cause Fowler syndrome^14^. Accumulating evidence suggests that defects in energy metabolism play a critical role in neurodegeneration. For example, the late-onset Alzheimer’s disease is associated with rare variants of TREM2^32^. TREM2-deficient microglia exhibit impaired mTOR activation and phagocytosis, which can be corrected with provision of an ATP precursor cyclocreatine^33^. Similarly, DNAJC30 interacts with ATP6, a component of ATP synthase, and its mutation leads to reduced ATP production and William syndrome^34^. Consistent with our findings, Castro-Gago et al. reported defects in ETC complexes III and IV in three patients from the same family with Fowler syndrome^35^. It is possible that reduced ATP synthesis and increased thermogenesis due to dysfunctional mutations in *Mfsd7c* could induce chronic cellular stress and compromise neuronal cell survival. Identification of MFSD7C as a heme-regulated switch between ATP synthesis and thermogenesis provides a basis to test this hypothesis.

Our study raises many new questions for future investigation. First, we show that MFSD7C predominantly resides in the mitochondria by subcellular fractionation and confocal microscopy, but whether it resides in the inner or outer mitochondrial membrane is currently unknown. This is an important question because the orientation of MFSD7C on the inner or outer membrane determines whether it senses heme in the lumen of mitochondria or in the cytosol. If MFSD7C resides in the inner membrane, MFSD7C likely senses heme in the lumen of mitochondria (Fig. 6a). In contrast, if MFSD7C resides in the outer membrane, it is likely involved in sensing cytosolic heme (Fig. 6b). Second, MFSD7C is a member of the solute carrier family and has been reported to transport heme based on some indirect evidence^19^. Although our study did not address this issue directly, our results that the NTD of MFSD7C binds to heme in vitro and is required to mediate the effect of heme in vivo are not incompatible with heme transport. However, there are several heme-regulated potassium channels that likewise sense heme via their N-terminal domains^36,37^, raising the possibility that MFSD7C is involved in the transport of calcium as initially suggested^22^, especially in light of its interaction with SERCA2b. Third, heme treatment of parental cells phenocopies the *Mfsd7c* knockout phenotype in uncoupling mitochondrial respiration. In our study, cells were treated with heme for 1 h prior to assaying for OCR, thermogenesis and co-IP, suggesting rapid and robust effect of heme. Although the concentration of heme used (5 to 40 μM) was likely higher than the labile heme concentration in the cytosol (<1 μM)^38–40^, a significant fraction of the exogenously added heme was likely bound by serum albumin and other proteins in the culture medium. Whether and how heme gains access to the cytosol or the lumen of the mitochondria to uncouple mitochondrial respiration remains to be examined. Fourth, binding of heme to the NTD disrupts MFSD7C interactions with ETC components and SERCA2b, leading to the stabilization of SERCA2b. Mechanistically, how this is achieved remains to be determined. Finally, we showed that MFSD7C regulates the uncoupling of mitochondrial respiration in response to heme in multiple cell types, including human monocytic cells, breast cancer cells, embryonic kidney cells, and mouse bone marrow-derived macrophages. MFSD7C transcript is detected at different levels in many cell types and tissues (http://www.immgen.org)^41^ and cellular heme is maintained through coordinated regulation of biosynthesis, uptake and degradation^42^. The combination of two variables, both MFSD7C and heme levels, could allow different cell types to dynamically regulate the outputs of mitochondrial respiration in order to meet their physiological need for ATP versus heat.

## Methods

### Purification of MFSD7C N-terminal domain (NTD)

Plasmid harboring GST-His-tag-SUMO-NTD (amino acids 1–84) was transformed into *Escherichia coli* Lemo21(DE3) strain (New England Biolabs), and grown overnight in 50 mL of LB supplemented with ampicillin (100 μg/mL) at 37 °C. The following day, 5 mL of overnight culture was diluted in 5 L of terrific broth media (TB) supplemented with ampicillin (100 μg/mL) at 37 °C and grown to O.D._600_ = 0.6. The expression of GST-His-tag-SUMO-NTD was induced with 0.5 mM IPTG for 16 h at 37 °C. Cells were harvested by centrifugation and washed once with ice-cold Milli-Q water. The washed pellet was resuspended in 50 mL of ice-cold buffer A (50 mM Tris-HCl pH 8.0, 500 mM NaCl, 0.5% Triton X-100) containing Protease Inhibitor Cocktail VII (RPI International), and the resuspension was frozen at −80 °C for no longer than one week. The resuspension was thawed at room temperature, and cells were lysed by sonication in the cold room. The lysate was cleared by centrifugation (20,000×*g* for 45 min) and the supernatant was incubated with 5 mL of Complete His-tag Purification Resin (Roche) pre-equilibrated with buffer A for 3 h while rotating in the cold room. The flow-through was discarded and the resin was washed twice with 25 mL of ice-cold buffer B (10 mM Tris-HCl pH 8.0, 100 mM NaCl). GST-His-tag-SUMO-NTD was eluted from the resin with 20 mL of buffer B containing 300 mM imidazole. Eluted fraction was supplemented with β-mercaptoethanol (f.c. 2 mM), and NTD was cleaved off from the rest of the protein with catalytic subunit of yeast Ulp1 (f.c. 1 μg/mL for 1 h in the cold room). After incubation with Ulp1, the mixture was flash-frozen in liquid nitrogen, lyophilized overnight, and resuspended in 10 mL of pure HPLC-grade water. This step eliminated volatile molecules such as β-mercaptoethanol, and selectively precipitated GST-His-tag-SUMO, Ulp1, and other contaminating proteins while it had no effect on the stability of water-soluble and intrinsically disordered NTD. Precipitate was cleared by centrifugation (20,000×*g* for 15 min) and the supernatant containing the NTD was transferred to fresh tubes. NTD was precipitated with isopropanol (f.c. 50% v/v) at −20 °C for 2 h, and centrifuged. The supernatant was discarded and the precipitate was dried in air at room temperature for 10 min. The precipitated NTD was resuspended in 5 mL of pure HPLC-grade water. Finally, the resuspended sample was applied to pre-equilibrated Superdex 75 10/300 gel-filtration column with filter-sterilized HPLC-grade water. Peak fractions containing NTD were pooled, lyophilized and resuspended in HPLC-grade water to desired concentration. Five liter of cells typically yield 15 mg of NTD, at least 95% pure according to SDS-PAGE and MALDI-TOF analysis. Mutant NTD carrying His30Ala/His36Ala/His48Ala/His54Ala/His66Ala mutations was purified the same way as the wild-type NTD.

### Peptide synthesis

Wild-type HP motif peptide (HPSALAQPSGLAHP) and mutant HP motif peptide (APSALAQPSGLAAP) were synthesized using solid-phase synthesis and purified using HPLC by the Biopolymers and Proteomics Core at the Koch Institute for Integrative Cancer Research.

### Heme absorbance-shift assay

Hemin was prepared fresh before each experiment. Approximately 20 mg of hemin (Sigma) was placed in a fresh tube and resuspended with 1 mL of DMSO. The hemin solution was slowly diluted while mixing with 1 mL of 2× buffer C (25 mM HEPES-NaOH pH 7.8, 10 mM NaCl), and aggregated hemin was eliminated by passing the solution through a 0.2 μm filter unit. Hemin concentration was determined by diluting the filtered solution with 1× buffer C 1:100 and using the extinction coefficient of 58,400 cm^−1^M^−1^ at 385 nm. For each reaction, the 200 μL reaction mix containing 100 μM hemin, 25 mM HEPES-NaOH pH 7.8, 10 mM NaCl, and various concentrations of wild-type or mutant NTD protein, was placed in a transparent 96-well plate. Absorbance intensity was measured using Tecan Infinite M200 Pro microplate reader, between 330 and 550 nm using 5 nm steps. The absorbance intensity for dissolved hemin was subtracted from absorbance intensity from hemin incubated with various concentrations of protein.

### Gel-filtration assay

Purified NTD (200 μM) was incubated with freshly prepared heme (150 μM) in a 1 mL reaction containing 5% DMSO, 25 mM HEPES-NaOH pH 7.8, and 10 mM NaCl. The solution was run over Superdex 75 (24 mL) gel-filtration column at 0.3 mL/min rate using AKTA-FPLC. Absorbance was monitored at 230, 380, and 415 nm. Two mililiter injection volume was subtracted from the final elution volume. Solution containing dissolved hemin in the reaction buffer without the NTD protein aggregated on top of the Superdex 75 column, thus this control was avoided in our experiments.

### Isothermal titration calorimetry (ITC)

ITC was performed using Microcal VP-ITC (Malvern). Freshly prepared 25 μM hemin solution (described above) in 10% DMSO, 25 mM HEPES-NaOH pH 7.8, was placed in the sample cell using bubble-free technique. The reference cell was filled with buffer containing 10% DMSO, 25 mM HEPES-NaOH pH 7.8 without hemin. The titration syringe was filled with 110 μM NTD protein in the matching buffer. The experiment was run following the manufacturer’s instructions using the following parameters: temperature was set to 25 °C, 27 total injections (the first injection was 1 μL with subsequent injections of 10 μL over 20 s), differential power was set to 10, delay was 60 s, and syringe was rotating at 307 rpm.

### Antibodies, cell lines and flow cytometry

Antibodies specific for MFSD7C (Catalog No. HPA037984) for Western blotting or immunofluorescence were purchased from Sigma. Antibodies specific for SERCA2b (Catalog No. ab2861) for immunofluorescence were purchased from Abcam. Antibodies specific for SERCA2b (Catalog No. 4388) for Western blotting were purchased from Cell Signaling Technology. Anti-Calnexin (Catalog No. ab13504) for ER localization was purchased from Abcam. Anti-Myc (Catalog No. 5605), anti-HA (Catalog No. 2367) and anti-FLAG (Catalog No. 2368) antibodies were purchased from Cell Signaling Technology. Human SERCA2b (Catalog No. 75188) plasmid was purchased from Addgene. Cell lines THP-1 (ATCC TIB-202), MH-S (ATCC CRL-2019), and 293FT^43^ were cultured following vendor instructions (37 °C, 5% CO_2_). FPT labeled cells were analyzed on BD-LSRII, collecting 20,000 live cells per sample. The data were analyzed using FlowJo.

### Mouse whole brain cellular fractionation analysis

Whole brain from C57BL/6 mice was isolated, resuspended in PBS supplemented with 10 mM EDTA, and passed through 40 μm Falcon cell strainer (VWR). The resuspension was centrifuged at 1200×*g* and washed twice more with PBS/10 mM EDTA. The pellet was resuspended in 35 mL of cold 1× MS Buffer (210 mM mannitol, 70 mM sucrose, 5 mM Tris-HCl pH 7.5, 1 mM EDTA) supplemented with 1% fatty acid-free BSA (Sigma). Cells were lysed using Dounce homogenizer with 10–15 strokes of the pestle, and the lysate was transferred to a 50 mL centrifuge tube and centrifuged at 1300×*g* for 5 min at 4 °C to precipitate nuclei and unbroken cells. The supernatant was transferred to a fresh 50 mL centrifuge tube and the nuclear precipitation step was repeated two more times. The supernatant was then centrifuged at 10,000×*g* for 15 min at 4 °C to precipitate mitochondria. The supernatant was saved for analysis (Sup), while the crude mitochondrial pellet was washed once more by resuspending in 35 mL of ice-cold 1× MS Buffer plus 1% BSA followed by centrifugation at 10,000×*g* for 15 min at 4 °C. Crude mitochondrial pellet was resuspended in 5 mL of 1× MS buffer, and mitochondria were used immediately for Western blot analysis (Mito). Western blot analysis was performed using the following antibodies: anti-MFSD7C (Sigma, Catalog No. HPA037984), anti-VDAC (Cell Signaling Technologies, Catalog No. 4661), anti-COX4I1 (Cell Signaling Technologies, Catalog No. 4850), anti-GAPDH (Cell Signaling Technologies, Catalog No. 5174), anti-NPM1 (Novus Biologicals, Catalog No. NB110-61646SS), anti-Calreticulin (Cell Signaling Technologies, Catalog No. 12238), anti-SERCA2b (Cell Signaling Technologies, Catalog No. 3010), and anti-LC3 (Cell Signaling Technologies, Catalog No. 2775).

### DNA plasmids for IP-MS, localization, co-IP, genome editing

To construct MFSD7C tagged with FLAG and Myc epitopes for immunoprecipitation-mass spectrometry (IP-MS), GFP-P2A fragment was amplified with the primers Bgl II-NHEI-GFP-F and BamHI-P2A-GFP-R. The fragment was digested using Bgl II/Bam HI and inserted to the Bam HI site of the pLKO.1 vector (Addgene Catalog No. 10878) to obtain pLKO.1-GFP-P2A-Puro vector. The MFSD7C-FLAG-Myc fragment was amplified from the murine MFSD7C plasmid (Origene Catalog No. MR208748) using the primers MFSD7C-SgfI-F and AC-Myc-DDK-MluI-KpnI-R. The fragment was digested with SgfI and then with KpnI. The fragment was cloned into the SgfI and KpnI sites of pLKO.1-GFP vector to yield pLKO.1-GFP-P2A-MFSD7C-FLAG-Myc (Supplementary Fig. 2a, b).

To construct MFSD7C-GFP fusion for localization study, MFSD7C fragment was amplified from the murine MFSD7C plasmid (Origene Catalog No. MR208748) with primers MFSD7C-SgfI-F and AC-Myc-DDK-MluI-KpnI-R and inserted into SgfI and MluI sites of pCMV6-AC-GFP vector (Origene Catalog No. PS100010) so that GFP is fused to the C-terminus of MFSD7C (Supplementary Fig. 2a, b).

To construct various vectors for co-immunoprecipitation, MFSD7C fragment was amplified from the murine MFSD7C plasmid (Origene Catalog No. MR208748) with the primers MFSD7C-SgfI-F and AC-Myc-DDK-MluI-KpnI-R and inserted in SgfI and MluI sites of pCMV6-AN-3HA (Origene Catalog No. PS100066) so that HA tag is introduced into N-terminus of MFSD7C. Murine Hmox1 (Catalog No. MR203944), Cyc1 (Catalog No. MR204721), Cox4i1 (Catalog No. MR218332), Ndufa4 (Catalog No. MR216909), ATP5h (Catalog No. MR201260), ATP5c1 (Catalog No. MR204152) genes were purchased from Origene and were tagged with FLAG at the C-terminus.

To construct vectors for MFSD7C knockout in cell lines, mCherry fragment was amplified using the primers Bam HI-P2A-mCherry-F and mCherry-WRPE-R. WRPE fragment was amplified with the primers mCherry-WRPE-F and PmeI-R. The mCherry-WRPE fragment was amplified with primers Bam HI-P2A-F and PmeI-R using the mixture of mCherry fragment and WRPE fragment as templates. The mCherry-WRPE fragment and Lenti-CRISPR-V2^44,45^ (Addgene Catalog No. 52961) were digested with Bam HI and PmeI and ligated to generate Lenti-CRISPR-V2-mCherry (Supplementary Fig. 3b). gRNA-1 and gRNA-2, specific for human MFSD7C, were inserted in BsmBI site of the Lenti-CRISPR-V2-mCherry vector. gRNA-3, specific for human MFSD7C, was inserted into the BsmBI site of Lenti-CRISPR-V2 vector (Addgene Catalog No. 52961).

To construct MFSD7C full length to complement 7CKO 4B8 cells, 3 MFSD7C fragments was amplified from the murine MFSD7C plasmid (Origene Catalog No. MR208748) by primer pairs MFSD7C-AsiSI-F/MFSD7C-KpnI-R, MFSD7C-KpnI-F/ MFSD7C-XmaI-R, MFSD7C-XmaI-F/ MFSD7C-MluI-R. The three fragments were Gibson assembled to pLKO.1-GFP-P2A-MFSD7C-Myc-DDK that was linearized by AsiSI and MluI. The correct constructs (pLKO.1-GFP-P2A-FL-MFSD7C-Myc-DDK) were validated by Sanger sequencing and used for Fig. 4a–f. To construct N-terminus deletion of MFSD7C for complementation of 7CKO 4B8 cells, 3 MFSD7C fragments was amplified from the murine MFSD7C plasmid (Origene Catalog No. MR208748) by primer pairs ΔNTD-MFSD7C-AsiSI-F/MFSD7C-KpnI-R, MFSD7C-KpnI-F/ MFSD7C-XmaI-R, MFSD7C-XmaI-F/ MFSD7C-MluI-R. The three fragments were Gibson assembled to pLKO.1-GFP-P2A-MFSD7C-Myc-DDK that was linearized by AsiSI and MluI. The correct constructs (pLKO.1-GFP-P2A-ΔN-MFSD7C-Myc-DDK) were validated by Sanger sequencing and used for Fig. 4a–f (Supplementary Fig. 2a, b).

All of the final constructs were confirmed by sequencing. See Supplementary Table 1 for a list of primers, and Supplementary Table 2 for a list of plasmids used in this study.

### Generation of lentiviral vectors and stable cell lines

The protocols for lentiviral production and transduction were as described^46^ (http://www.addgene.org/tools/protocols/plko/). Briefly, the plasmids of lentivector, psPAX2 (packaging, Addgene Catalog No. 12260), and pMD2.G (envelope, Addgene Catalog No. 12259) were transfected into 293T cells for lentiviral production. The lentivirus was tittered and used to transduce the target cells. Transduced cells were purified by flow cytometry using the encoded fluorescence proteins in the lentivectors or were selected by puromycin using the resistance gene encoded in the lentivector. Murine alveolar macrophage cell line MH-S was transduced with pLKO.1-GFP-P2A-Puro and pLKO.1-GFP-P2A-MFSD7C-FLAG-Myc. GFP positive cells were sorted and expanded. Cell lysates were then used for immunoprecipitation, followed with mass spectrometry.

THP-1 cells were transduced with lentiviruses expressing mCherry, Cas9, and MFSD7C guide RNA-1 or RNA-2 (Supplementary Fig. 3b, d). mCherry-positive cells were cloned by single cell sorting into a 96-well plate. Deletion of MFSD7C in the clones was determined by PCR analysis followed by sequencing. Specifically, the genomic DNA of the targeted regions was amplified with the specific primers F1/R1, F2/R2, F3/R3 for sequencing, respectively (Supplementary Fig. 3d). Two clones 3D12 and 4B8, one each derived from gRNA-1 and gRNA-2, were identified to have deletions in MFSD7C genomic DNA with no detectable RNA transcript. To ensure the complete MFSD7C knockout, these clones were subject to another round of CRISPR-Cas9-mediated gene editing using lentivirus expressing puromycin resistant gene, Cas9 and MFSD7C gRNA-3 (Supplementary Fig. 3c, d). Puromycin-resistant cells were again cloned by single cell sorting. A total of 4 clones were identified to have the genomic deletion and no detectable wildtype genomic DNA and transcript (Supplementary Fig. 3e). The four clones are collectively referred to as 7CKO clones/cells. CRISPR-Cas9-mediated MFSD7C targeting in MCF7 and 293 T cells were done in the same manner.

### Immunoprecipitation and LC-MS/MS

Murine alveolar macrophage cell line MH-S, which expresses MFSD7C (Supplementary Fig. 2c), was transduced with lentivirus expressing GFP alone or GFP plus murine MFSD7C tagged with Myc and FLAG epitopes. The GFP positive cells were sorted and expanded. Two hundred million cells per sample were lysed in 5 mL of cold Lysis Buffer, containing 20 mM Tris-HCl, pH 7.4, 150 mM NaCl, 0.1% NP-40, 10% glycerol, proteinase inhibitor (Sigma Catalog No. 4693132001), and phosphatase inhibitors (Sigma Catalog No. 4906845001), and homogenized. The lysates were centrifuged at 30,000×*g* for 10 min, and the supernatants were further centrifuged at 30,000×*g* for 20 min. The clear supernatants were incubated with M2 magnetic beads conjugated with anti-FLAG antibody (Sigma Catalog No. M8823) for 2 h and eluted by 3× FLAG peptide. The elutes were incubated with magnetic beads conjugated to anti-Myc antibody (Cell Signaling Technology Catalog No. 5698) for 2 h. The beads were washed in Lysis Buffer and balanced by PBS. The immunoprecipitates were washed three times with 100 mM NH_4_HCO_3_. Proteins were reduced (10 mM dithiothreitol, 56 °C for 45 min) and alkylated (50 mM iodoacetamide, room temperature in the dark for 1 h). Proteins were subsequently digested with trypsin (sequencing grade, Promega), at an enzyme/substrate ratio of 1:50, at room temperature overnight in 100 mM ammonium acetate, pH 8.9. Trypsin activity was quenched by adding formic acid to a final concentration of 5%. Peptides were desalted using C18 SpinTips (Protea) then lyophilized and stored at −80 °C.

Peptides were loaded on a pre-column and separated by reverse phase HPLC (Thermo Easy nLC1000) over a 140-min gradient before nanoelectrospray using a QExactive mass spectrometer (Thermo). The mass spectrometer was operated in a data-dependent mode. The parameters for the full scan MS were: resolution of 70,000 across 350–2000 *m/z*, AGC 3e^6^, and maximum IT 50 ms. The full MS scan was followed by MS/MS for the top 10 precursor ions in each cycle with a NCE of 28 and dynamic exclusion of 30 s. Raw mass spectral data files (.raw) were searched using Proteome Discoverer (Thermo) and Mascot version 2.4.1 (Matrix Science). Mascot search parameters were: 10 ppm mass tolerance for precursor ions; 0.8 Da for fragment ion mass tolerance; 2 missed cleavages of trypsin; fixed modification was carbamidomethylation of cysteine; variable modification was methionine oxidation. Only peptides with a Mascot score greater than or equal to 25 and an isolation interference less than or equal to 30 were included in the data analysis. Potential interacting proteins are identified in the experimental sample after removal of proteins in the control sample and common contaminating proteins.

The mass spectrometry proteomics data have been deposited to the ProteomeXchange Consortium via the PRIDE^47^ partner repository with the dataset identifier PXD021016.

### Co-transfection and immunoprecipitation

For co-IP, HA-tagged MFSD7C was co-transfected with FLAG-tagged HMOX1 (Origene Catalog No. MR203944), CYC1, COX4I1, NDUFA4, ATP5h, ATP5c1 into 293FT cells using TransIT®-LT1 Transfection Reagent (Mirus). Thirty-six hours after transfection, the cells were lysed using cold Lysis Buffer containing 20 mM Tris-HCl (pH 7.4), 150 mM NaCl, 0.1% NP-40, 10% glycerol, proteinase inhibitor (Sigma Catalog No. 4693132001), and phosphatase inhibitors (Sigma Catalog No. 4906845001). The clear supernatants from the lysate were incubated with M2-mangnetic beads conjugated with anti-FLAG antibody (Sigma Catalog No. M8823) for 2 h at 4 °C. Then the beads were washed twice and eluted by the 3× FLAG peptides (Sigma Catalog No. F4799).

To determine the effect of heme on MFSD7C interactions with ETC components, HMOX1 or SERCA2b, 293FT cells were transiently transfected with HA-tagged murine MFSD7C and FLAG-tagged murine CYC1, NDUFA4, COX4i1, ATP5h, ATP5c1, HMOX1 or SERCA2b. 35 h later, co-transfected cells were incubated with DMSO (vehicle) or 10 μM or 40 μM of heme for 1 h before lysis. Cell lysates were precipitated with anti-HA antibody, eluted with HA peptide, further precipitated with anti-FLAG antibody, eluted with FLAG peptide, and then subjected to Western blotting with anti-HA and anti-FLAG antibodies. Cells were treated with proteasome inhibitor MG132 for co-IP between MFSD7C and SERCA2b.

### Endogenous protein extraction

THP-1 cells were lysed in RIPA buffer (25 mM Tris•HCl pH 7.4, 150 mM NaCl, 1% NP-40, 1% sodium deoxycholate, 0.1% SDS) with proteinase and phosphatase inhibitors (Sigma Catalog No. 4693132001 and 4906845001). The clear supernatants were used for Western blotting^48^.

### Imaging analysis of MFSD7C localization

For MFSD7C localization, 293FT cells over-expressing GFP-tagged or mCherry-tagged MFSD7C were grown on coverslips in tissue culture and stained for mitochondria using 100 nM MitoTracker® Deep Red FM (Thermo-Fisher Catalog No. M22426) for 20 min in serum-free medium, per manufacturer’s protocol. Cells were fixed using 3.5% paraformaldehyde (in 1× PBS, pH 6.7) for 10 min and permeabilized with 0.5% Triton-X in 1× TBS-BSA (10 mM Tris HCl pH 7.5, 150 mM NaCl, 1% BSA, 0.1% NaN_3_) for another 10 min. Anti-human HLA-A, B, C (Biolegend W6/32) was added at a 1:1000 dilution in 1× TBS-BSA + 0.1% Triton-X for 1 h at room temperature. Anti-mouse Alexa 647 (Life Technologies Catalog No. A31571) at a 1:2000 dilution was added to DAPI in 1× TBS-BSA and incubated with the cells for 1 h.

Coverslips were attached to glass slides using ProLong® Diamond Antifade Mountant with DAPI (Thermo-Fisher, Catalog No. P36962) and imaged using a Nikon A1R Ultra-Fast Spectral Scanning Confocal Microscope using Elements software. Images were taken in z-stacks of 0.2 μm and flattened using the max projection function in ImageJ.

### Measurements of OCR, ECAR, and MMP

Oxygen consumption rate (OCR) and extracellular acidification rate (ECAR) were measured using XF96e Seahorse Extracellular Flux Analyzer per manufacturer’s protocol. To increase adherence of suspension cells, Seahorse plates were coated with Corning® Cell TAK (Catalog No. 354240). THP-1 and 7CKO cells were then attached to the plate according to the manufacturer’s instructions. Cells were incubated in complete RPMI media with or without 40 µM heme. Changes in oxygen consumption were measured following treatment with oligomycin (5 µM), FCCP (2 µM), and rotenone (1 µM) plus antimycin A (1 µM). For BMDM, 5 × 10^5^ cells/well were plated in 100 μL of BMDM media, 24 h before the start of the assay. For OCR measurements, BMDM media was replaced with 180 μL of Seahorse XF Base Medium supplemented with 10 mM D-glucose, 1 mM sodium pyruvate, and 1 mM L-glutamine. For ECAR measurements, BMDM media was replaced with 180 μL of Seahorse XF RPMI Medium pH 7.4 supplemented with 2 mM L-glutamine. The Glycolysis Stress Test was performed using D-glucose, rotenone/antimycin A, and 2-deoxy-D-glucose at 10 mM, 0.5 μM, and 50 mM final concentration, respectively.

Mitochondrial membrane potential was measured using Abcam kit (Catalog No. ab113852). Briefly, BMDM, THP-1 and 7CKO cells were not treated or treated with 40 µM heme for 1 h. The cells were incubated with mitochondrial membrane potential indicator, 200 nM TMRE (tetramethylrhodamine, ethyl ester) for 20 min. The mean fluorescence intensity of TMRE were determined by flow cytometry.

### Measurement of cellular energy charge (ATP/ADP ratio)

ATP/ADP ratio was measured using ADP Assay Kit (Sigma Catalog No. MAK133-1KT) following manufacturer’s protocol. For THP-1 cells, 24 h before the experiment, cells were resuspended in fresh complete RPMI media. For heme treatment, 1 mL of treated cell suspension was incubated for 1 h at 37 °C cell culture incubator, then centrifuged and resuspended in 1 mL of fresh warm complete RPMI media in order to wash off excess heme, which interferes with the assays. Ten microliter of cell suspension per well (approximately 10,000 cells total) was placed in a white 96-well plate, lysed with 90 µL of ATP Buffer, and incubated with gentle shaking at room temperature for 10 min. Relative ATP amount was directly measured using luminescence. Then, 5 µL of ADP Enzyme mix was added to each well and the plate was incubated with light shaking for 3 min. Relative amount of ADP + ATP was measured using luminescence. ADP amount was calculated by subtracting the ATP signal from ADP + ATP signal. To get ATP/ADP ratio, ATP signal was divided by the calculated ADP signal. For BMDMs, the assay was performed following the same protocol but using 20,000 cells per well.

Alternatively, ATP, ADP, and AMP levels were measured using targeted metabolomic analysis at the Whitehead Institute Metabolite Profiling Core Facility. Briefly, 100,000 cells (parental THP-1 cells and 7CKO clone B11, 3 independent experiments each) were centrifuged, and resuspended in 500 µL of cold 0.9% NaCl. Metabolites were extracted with the addition of 600 µL LC/MS-grade cold methanol containing internal standards, and the mixture was vortexed for 2 min. Then 300 µL LC/MS grade water was added to each tube, followed by 400 µL cold chloroform. The mixture was vortexed again in the cold room and then spun at 16,000×*g* in a microcentrifuge. The top layer containing polar metabolites was transferred to a clean tube and the sample was dried using speedvac. Ultra-pressure liquid chromatography was performed using pHILIC column on Dionex UltiMate 3000 and mass-spectrometry was performed using Thermo Scientific QExactive Orbitrap instruments.

### Synthesis of fluorescent polymeric thermometer

4-N,N-Dimethylaminosulfonyl-7-fluoro-2,1,3-benz-oxadiazole (DBD-F) was purchased from TCI Chemicals. N-n-propylacrylamide (NNPAM) and N-ethylacrylamide (NEAM) were purchased from AstaTech. N, N’-dimethylethylenediamine, acryloyl chloride, triethylamine (TEA), (3-acrylamidopropyl) trimethylammonium chloride (APTMA Cl), azobisisobutyronitrile (AIBN) and other solvents were purchased from MilliporeSigma. All commercial acrylamide monomers were passed through a basic alumina column to remove inhibitors before polymerization. Other reagents were used as purchased.

The synthesis was slightly modified from the protocol in the literature^49^. Briefly, 100 mg DBD-F is dissolved in 5 mL anhydrous acetonitrile, then added dropwise into a stirring vial containing 1.3 mL N, N’-dimethylethylenediamine. The mixture was allowed to react for 15 min at room temperature. The reaction mixture was condensed with rotatory evaporation and purified with silica gel liquid chromatography using eluent dichloromethane:methanol from 10:1 to 5:1, fractions were collected and monitored with thin layer chromatography. DBD-NMe(CH_2_)_2_NHMe was obtained as an orange liquid.

One thirty microgram DBD-NMe(CH_2_)_2_NHMe was dissolved in 5 mL of anhydrous acetonitrile mixed with 58 μL of TEA and cooled on ice. Forty-four microliter acryloyl chloride was dissolved in 8 mL of anhydrous acetonitrile, cooled on ice and then added dropwise into the reaction mixture. The reaction was allowed to proceed for 1.5 h at room temperature then condensed with rotatory evaporation and purified with silica gel liquid chromatography using eluent ethylacetate:hexane 3:1. Fractions were collected and monitored with thin layer chromatography. DBD-AA was obtained as an orange powder (Supplementary Fig. 4a).

The polymer synthesis was modified from the protocols described in the literature^23,50^. Briefly, 4.1 mg AIBN, 20 mg DBD-AA, 41 mg APTMA Cl, 565 mg NNPAM (for fluorescent polymeric thermometer) or 495 mg NEAM (for control polymer that is not temperature sensitive), 5 mL DMF and a stir bar were added into a clean schlenk flask. The flask was sealed and purged with nitrogen for 30 mins at room temperature to remove dissolved oxygen. The flask was then immersed in 60 °C oil bath to initiate the polymerization. After 12 h of reaction, the reaction mixture was precipitated in cold ethyl ether (0 °C) and redissolved in DMF for three times, then dried in vacuo overnight for use (Supplementary Fig. 4b).

### Measurements of cellular thermogenesis

Three methods were used to measure cell thermogenesis. In the first approach, we used thermocouple. Three million THP-1 and 7CKO cells were re-suspended in 100 μL media (RT, room temperature) and transferred to PCR tubes tightly fitted in a thermally insulating enclosure. Then the temperature change rate of media (ΔT_m_/Δt) was monitored real-time by type T-Type Thermocouples (Omega, Catalog No. 5SC-TT-T-3036) immersed in the liquid media. An analog to digital converter (National Instruments 24-bit Thermocouple ADC) was used to obtain and store the thermocouple data via a custom Labview interface (sampling rate: 500 ms). Every measurement was compared to the free liquid media.

In the second approach, we used fluorescent polymeric thermometer (FPT)^23^, which we synthesized in-house (see above). Parental and knockout THP-1 cells were mixed together, washed and incubated with FPT in 5% w/v glucose solution for 6 h. The cells were washed with PBS twice and reseeded in dishes with glass bottom that was coated with poly-lysine. The cells were imaged under Confocal Laser-Scanning Microscopy at 37 °C. Alternatively, THP-1, MCF7, 293T, 7CKO, *SERCA2b*^*−/−*^ cells were incubated with FPT and control polymer overnight and followed with vehicle or hematin treatment for 1 h. Then the fluorescence intensities were determined by a flow cytometer.

In the third approach, we used commercial cellular fluorescent thermoprobe dye (Funakoshi Catalog Number: FDV-0005). Briefly, one day before the assay, 2 × 10^5^ BMDMs were plated in a 48-well non tissue-culture treated polystyrene plate in 200 μL of BMDM media. BMDM media were aspirated and cells were washed once with Loading Solution (5% (w/v) aqueous glucose solution supplemented with 10 mM EDTA). Next, 200 μL of Loading Solution supplemented with 50 ng/μL Cellular Thermoprobe Dye was added to the well and loading of the dye was performed in a 33 °C, 5% CO_2_ cell culture incubator for 7.5 min, which simultaneously lifts BMDMs from the well. In the next 2.5 min, the plate was removed from the incubator, cells were resuspended by pipetting and moved to a PCR tube and combined with 22 μL of 10× PBS/DAPI buffer. The mixture in the PCR tube was then placed in a thermocycler with a preset temperature, incubated for 5 min, and immediately analyzed using flow cytometry (BD LSR II). Cells are gated based on size (singlets) and DAPI (live cells). 15,000 FITC-positive cells were collected for the analysis. Special care was taken with regards to timing because the loading of the Cellular Thermoprobe Dye is dependent on the amount of time the cells are incubated with the dye. Different filters were used to measure loading and temperature sensitivity due to properties of Cellular Thermoprobe Dye. For loading measurements, excitation at 488 nm with 515/20 emission filter was used, while thermosensitive analysis was performed using excitation at 488 nm with 530/30 emission filter.

### Generation of *Mfsd7c* mutant mice

C57BL/6 N ES cell clone with floxed exon 2 of *Mfsd7c* was purchased from EuMMCR (European Mouse Mutant Cell Repository, ES cell Clone ID: HEPD0572_8_F01). The ES cells were transfected with plasmids encoding FLP to remove neomycin resistance cassette. The G418 sensitive ES cells with properly floxed exon 2 of *Mfsd7c* were confirmed using PCR and Sanger Sequencing. The ES cells were injected into blastocysts and then transferred into pseudopregnant mice at the Koch Institute Swanson Biotechnology Center. Germline mutant mice were identified based on the coat color and *Mfsd7c*^*wt/fl*^ heterozygous mice were interbred to generate homozygous *Mfsd7c*^*fl/fl*^ mice. *Mfsd7c*^*fl/fl*^ mice were bred with *LysM-Cre* mice (the Jackson Laboratory, Stock No: 004781) to generate myeloid-specific *Mfsd7c* knockout. Mice were maintained in the animal facility at the Massachusetts Institute of Technology (MIT). All animal studies and procedures were carried out following federal, state, and local guidelines under an IACUC-approved animal protocol by Committee of Animal Care at MIT.

### Mouse genomic DNA extraction, genotyping, and qPCR

A small piece of mouse tail was cut using scissors, placed in 500 μL of Genomic DNA Extraction Buffer (100 mM Tris-HCl pH 8.0, 200 mM NaCl, 5 mM EDTA, 0.2% SDS, 0.5 mg/mL Proteinase K) and digested overnight at 55 °C. Digested tissue was centrifuged at 13,000×*g* for 5 min, and the cleared supernatant was transferred to a fresh 1.5 mL tube. Genomic DNA was precipitated from the supernatant with isopropanol (50% v/v), and centrifuged at 13,000×*g* for 2 min. Supernatant was discarded, and the precipitate was washed with 1 mL of 70% ethanol. Ethanol was discarded and the precipitate was left to dry at room temperature for 5 min. DNA was resuspended in 200 μL of nuclease-free ddH_2_O. Genomic DNA from bone marrow-derived macrophages was isolated from 5 × 10^5^ cells using the same protocol. Primer set #1 and #2 were used to genotype wild-type, floxed and deleted alleles, and LysM-Cre primers were used to detect the presence of Cre recombinase (see Supplementary Table 1 for primer sequences).

For qPCR analysis, 10^6^ bone marrow-derived macrophages were rinsed with PBS, lysed in RLT buffer (Qiagen), and flash-frozen in liquid nitrogen. After thawing on ice and passing through a 27 G needle multiple times, RNA was isolated using Qiagen’s RNEasy Mini kit. cDNA was synthesized using Superscript IV (Invitrogen) and random hexamers, followed by RNA removal using *E. coli* RNase H for 20 min at 37 °C. cDNA was diluted tenfold and qPCR was performed in triplicate using 4 µL diluted cDNA, 0.5 µL 5 µM forward primer, 0.5 µL 5 µM reverse primer, and 5 µL 2× SYBR Green Master Mix (Roche) per well in a 96-well plate (see Supplementary Table 1 for primer sequences). A Roche Lightcycler 480 instrument was used measuring amplification for 45 cycles using Roche’s SYBR Green protocol, after which melting temperatures and crossing points were assessed and quantified.

### Differentiation of bone marrow-derived macrophage (BMDM)

*Mfsd7c*^*fl/fl*^ and *Mfsd7c*^*−/−*^ (*Mfsd7c*^*fl/fl*^ *LysMCre*^*+/+*^) C57BL/6 J mice were euthanized using CO_2_ asphyxiation. Femoral bones were removed and cleaned and the bone marrow was flushed out using 5 mL of cold DMEM media. Bone marrow cells were collected by centrifugation (1200×*g* for 5 min at 4 °C) and resuspended in 4 mL of ACK lysis buffer. After incubation at room temperature for 5 min, ACK buffer was neutralized by the addition of 11 mL of cold DMEM media and cell suspension was centrifuged at 1200×*g* for 5 min at 4 °C. Cells were resuspended in DMEM media containing 10% FBS, 20% L929-conditioned media, 2 mM L-glutamine, 2 mM pyruvate, non-essential amino acids (100 μM each), 0.55 mM 2-mercaptoethanol, penicillin/streptomycin), passed through a 40 μm Falcon cell strainer (VWR) to remove large aggregates, and counted. Bone marrow cells were seeded in 10 cm non tissue-culture treated plates at 10 million cells per plate in 10 mL of BMDM media. Two days later, additional 10 mL of BMDM media was added. On day 4 and day 6, old media was removed and 10 mL of fresh media was added. On day 7, fully differentiated BMDM were lifted in phosphate-buffered saline supplemented with 10 mM EDTA for 5 min at 37 °C, centrifuged and resuspended in BMDM media to a proper density for further experimentation.

### Figure illustrations

Certain images were used from the Library of Science and Medical Illustrations to create illustrations in Fig. 6a, b, and Supplementary Fig. 7a,c^51^. In Fig. 6a, b MFSD7C protein image was created using PyMOL^52^. Supplementary Fig. 1a was created using WebLogo^53^.

### Statistics and reproducibility

Data were statistically analyzed by one-way or two-way ANOVA, or by Student’s *t*-test using GraphPad Prism as described in Figure legends. The bar and error bar were defined as mean and standard deviation or standard error of the mean (SEM) and described in the Figure legends.

## Supplementary information

Supplementary Information

Peer Review File

## Data Availability

The authors declare that data supporting the findings of this study are available within the paper and its Supplementary Information files. Detailed information and materials are available upon request. The raw Western blotting data (for Figs. 1d–f, 2k, 4g, 5a–d, and Supplementary Figs. 3a, 6a, 7g, 9b), raw image data of Figs. 1g, 2g, 4e, Supplementary Figs. 2d, e, 5c, 9c, e, 10a, b, d and f are uploaded and available from Figshare^54–56^. The raw proteomics data for Table 1 are available via ProteomeXchange with identifier PXD021016^47,57,58^. All other data supporting the findings of this study are available from the corresponding author upon request. Source data are provided with this paper.

## Source data

Source Data
