# Supplementary material for: MFSD7C switches mitochondrial ATP synthesis to thermogenesis in response to heme
Source: Nat Commun. 2020 Sep 24;11:4837. doi: 10.1038/s41467-020-18607-1 (PMC7515921; doi:10.1038/s41467-020-18607-1)
Supplement: Supplementary file 1 — Supplementary Information [file 41467_2020_18607_MOESM1_ESM.pdf]

SUPPLEMENTARY INFORMATION

MFSD7C Switches Mitochondrial ATP Synthesis to Thermogenesis in Response to Heme

Li et al.

This file includes:

Supplementary Figures 1-10

Supplementary Table 1

Supplementary Table 2

Supplementary Figure 1.

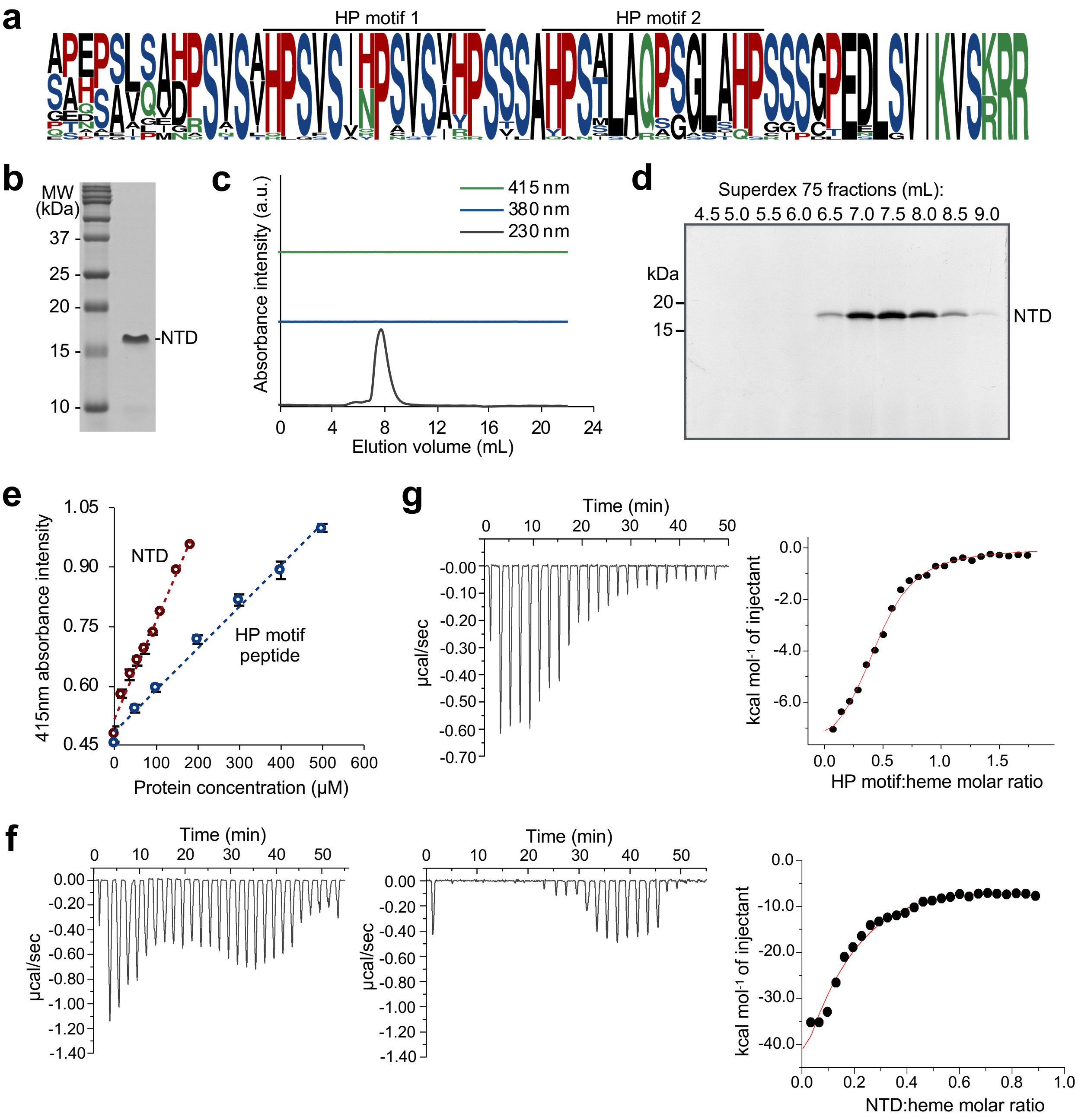

Supplementary Figure 1. The N-Terminal domain of MFSD7C contains heme-binding motifs. a NTD motif logo generated from the alignment of 29 mammalian sequences. Two putative heme-binding motifs containing histidine-proline residues (HP motif) are marked. **b** SDS-PAGE analysis of purified human NTD, stained with Coomassie blue. **c** Superdex 75 gel filtration chromatograms of NTD alone at absorbance at 230 nm, 380 nm and 415 nm. **d** SDS-PAGE analysis of the peak gel filtration fractions from Figure 1a stained with Coomassie Blue. **e** Plot of 415 nm Soret band absorbance versus molar ratio of NTD or HP motif peptide to heme (n=3 independent experimentsm, error bars represent standard deviation). **f** Isothermal titration calorimetry analysis of NTD binding to heme: NTD titrated into heme (left), NTD titrated into blank buffer (middle), and 3-site sequential binding model fit to isotherm of NTD binding to heme subtracted background (right). **g** Isotherm titration calorimetry analysis of the HP motif binding to heme (left) and one-site binding model fit (right).

Supplementary Figure 2.

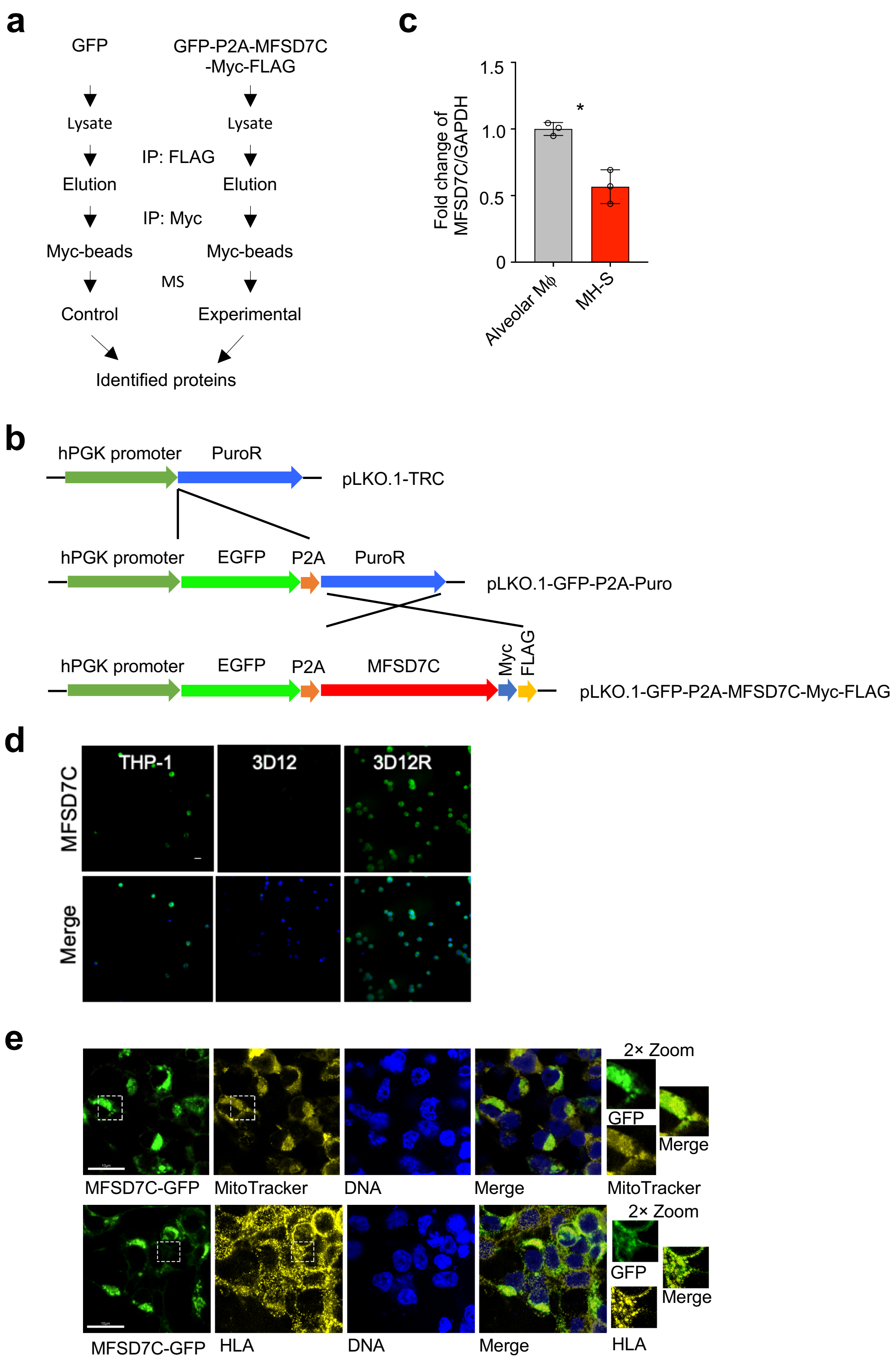

Supplementary Figure 2. MFSD7C interacts with mitochondrial ETC components. **a** Scheme for the isolation and identification of the MFSD7C-interacting proteins by IP-MS. **b** Schematics of control (GFP) and experimental (GFP-MFSD7C-Myc-FLAG) vectors. **c** Fold changes in GAPDH-normalized MFSD7C transcript levels in freshly purified murine CD11c^{+} Siglec-F^{+} CD11b^{lo} alveolar macrophages (n=3 mice) and murine alveolar macrophage cell line MH-S (n=3 biologically independent samples). Data are presented as mean value ± standard deviation. *p<0.01 by unpaired t-test. **d** Polyclonal antibodies to the C-terminal domain of MFSD7C are specific. Parental THP-1 cells, clone 3D12 of *Mfsd7c* knockout in THP-1 cells, and 3D12 reconstituted with a full length of MFSD7C (3D12R) were stained with polyclonal antibodies specific to the C-terminal domain of MFSD7C and DAPI. Shown are images of endogenous MFSD7C (top row) and merged images with DAPI (bottom row) in parental THP-1 cells (left column), 3D12 clone (middle column), and 3D12R (right column). Representative data from three separate experiments are shown. Scale bar, 10 μm. **e** Localization of MFSD7C. MFSD7C-GFP fusion protein was expressed in 293T cells and cells were stained with anti-HLA antibody, MitoTracker and DAPI and visualized by confocal microscopy. Most GFP signals co-localized with MitoTracker signals, but some GFP signals also co-localized with anti-HLA staining on the plasma membrane. Shown are representative data from three separate experiments. Scale bar represents 10 μm, and merged images are a 2x magnification of the selected area.

Supplementary Figure 3.

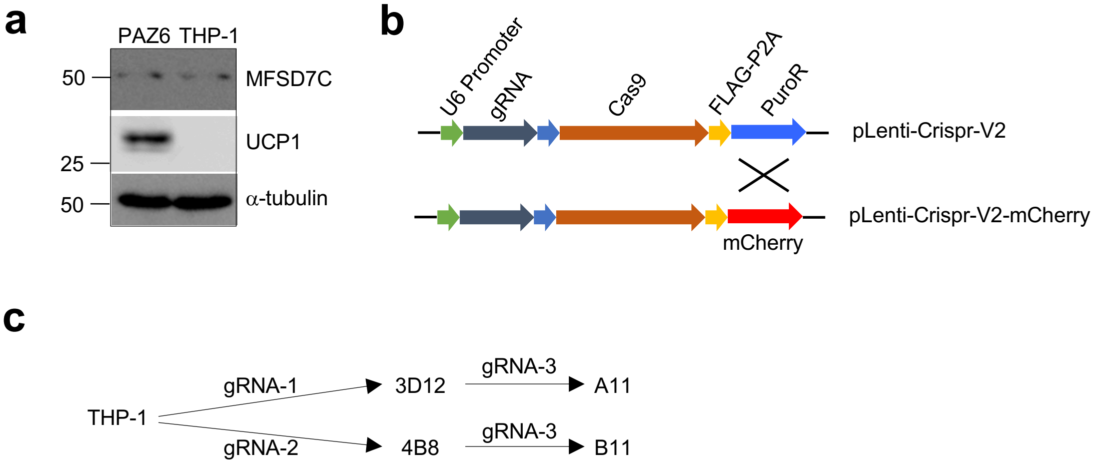

d

Clone name	Sequences of gRNA	Sequences of PCR primers
3D12	gRNA-1: CTCGTCCCGGTCTTCAATGT	F1: CCAGATATGGGAGTAGAGGA<br>R1: ACAAAGTGCATAGGACCAGC
4B8	gRNA-2: CTCTTCTGAATCGCATGGTG	F2: TGTCCAGGACTTCTACTGAC<br>R2: GCTCAGAAACACTAACTAGC
A11/B11	gRNA-3: GAACCTAAGTATGAGGCATC	F3: CTGGGTGACAGAGCGAGACT<br>R3: ACCAACAGGCATTTGTCAGA

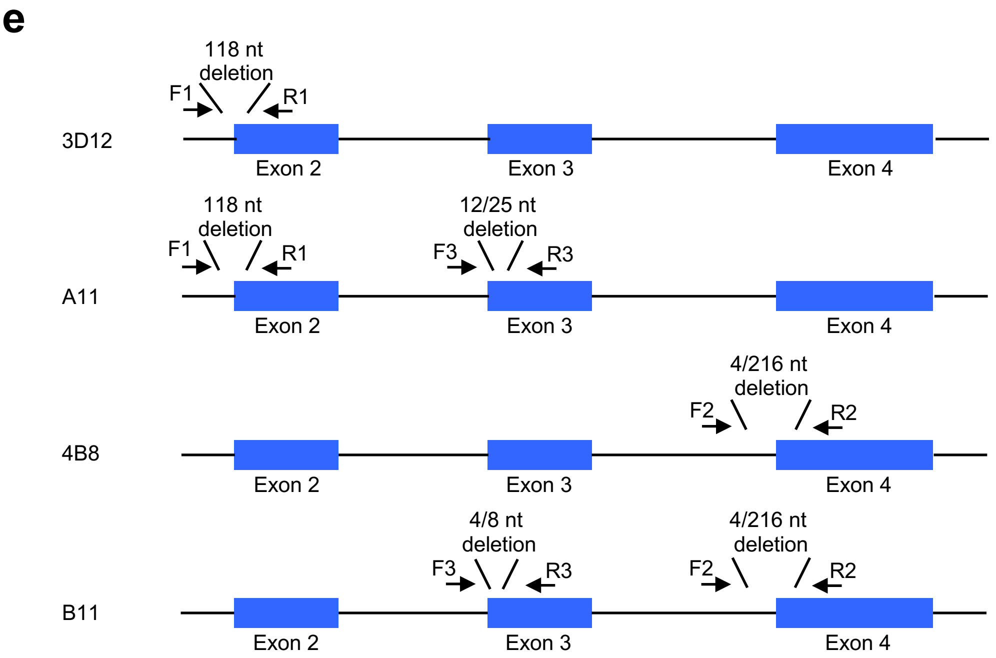

Supplementary Figure 3. Construction of MFSD7C knockout THP1 cells by CRISPR-Cas9-mediated gene editing. a Western blotting analysis of MFSD7C and UCP1 proteins in THP-1 cells and PAZ6 cells, an immortalized human brown pre-adipocyte line. The cell lysates were blotted with a polyclonal antibody to the C-terminus of MFSD7C and a monoclonal antibody to UCP1 or 〈-tubulin. **b** Scheme of lentiviral vectors. **c** Scheme for constructing MFSD7C knockout THP-1 cells, showing two rounds of CRISPR-Cas9-mediated gene editing. Two MFSD7C specific guide RNA (gRNA) sequences were cloned into Lenti-CRISPR-V2-mCherry vector. The plasmids and lentivirus packaging plasmids were transfected into 293FT cells to produce lentiviruses expressing Cas9 and guide RNA (gRNA). THP-1 cells were transduced with these lentiviruses and cloned by sorting for mCherry expressing cells. The first round of editing produced clones 3D12 and 4B8, and the second round produced clone A11 from 3D12 and clone B11 from 4B8 by lentivirus expressing gRNA-3 sequences, Cas9 and the puromycin resistance protein. **d** Sequences of gRNA and PCR primers used. **e** Illustration of deletions in genomic DNA of different clones as determined by PCR amplification and sequencing.

Supplementary Figure 4.

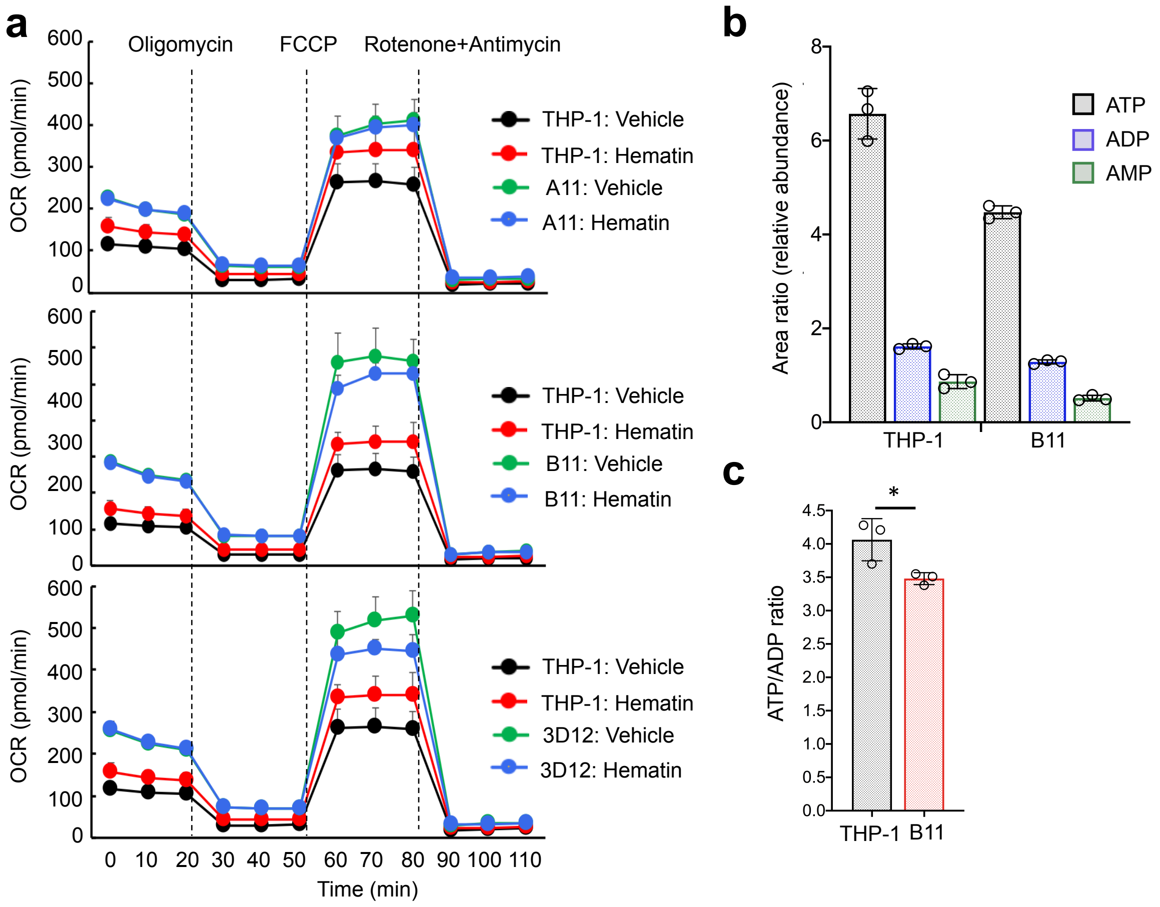

Supplementary Figure. 4. MFSD7C knockout stimulates OCR and thermogenesis. **a** Representative graphs of OCR output of THP-1, A11, B11, and 3D12 cells with or without hematin treatment for 1 hour and their responses to oligomycin, FCCP, and rotenone plus antimycin A from XF96e analyzer. **b** Targeted metabolomic analysis of ATP, ADP, and AMP levels. **c** ATP/ADP ratio of parental THP-1 and 7CKO clone B11 cells (100,000 cells each, n=3 independent experiments). Data are presented as mean value ± standard deviation. P-values were calculated using unpaired t-test (*$p<0.05$).

Supplementary Figure 5.

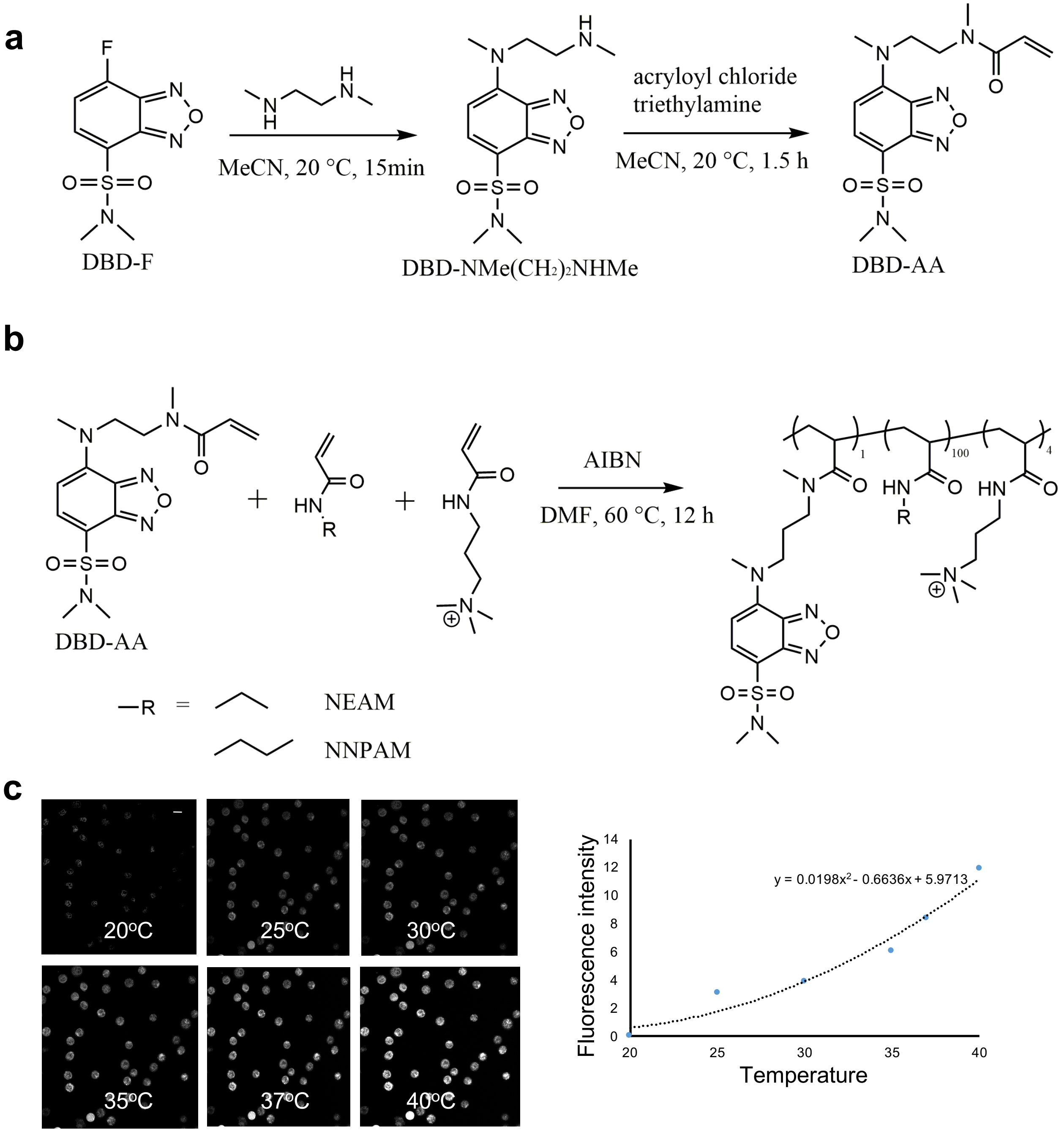

Supplementary Figure. 5. Measuring thermogenesis using fluorescent polymeric thermometer (FPT). **a** and **b** Scheme of FPT synthesis (see Methods for detail). **c** FPT fluorescence intensity at different temperature in THP-1 cells (left). Plot of fluorescence intensity versus temperature with curve fitting equation (right).

Supplementary Figure 6.

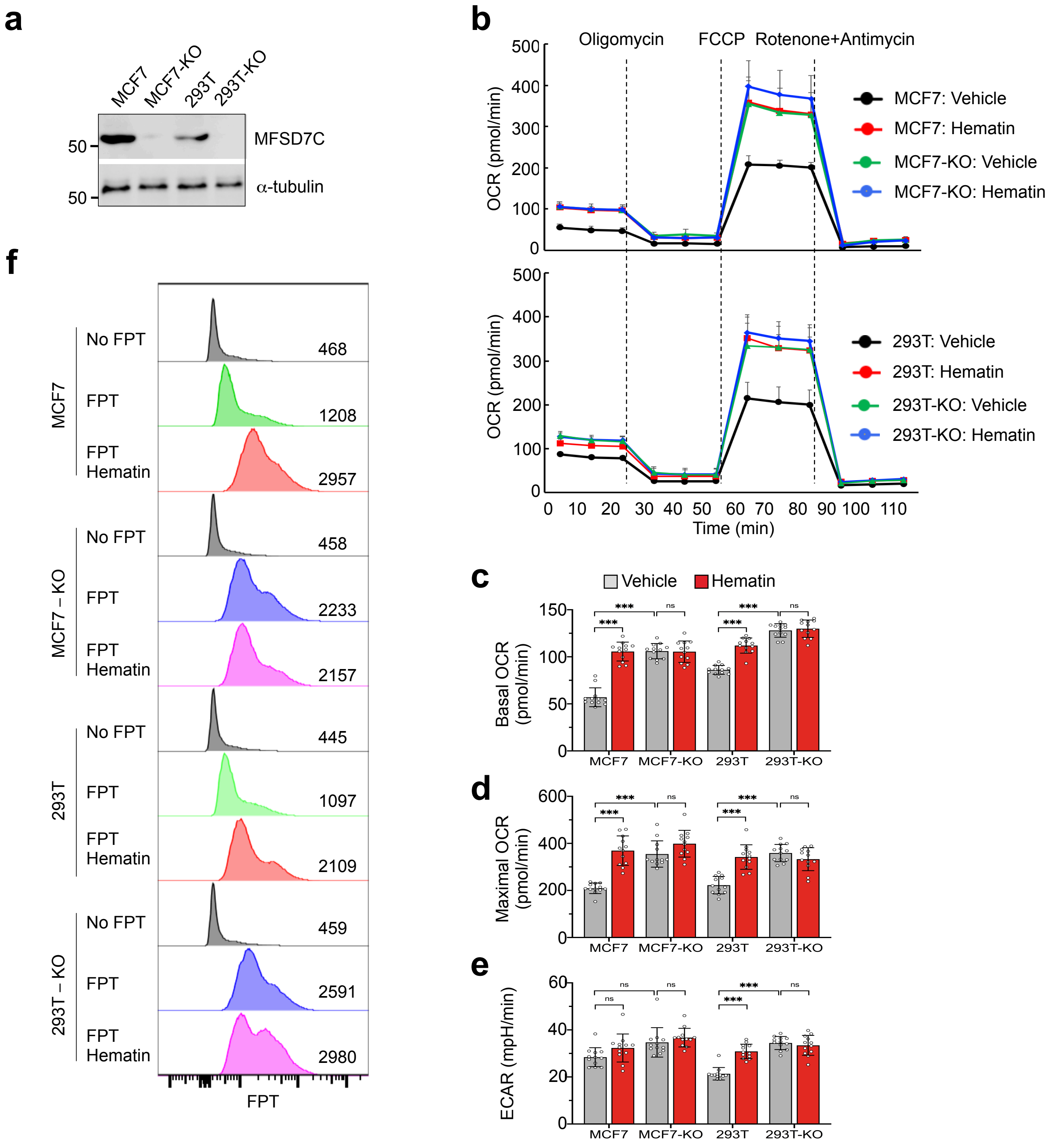

Supplementary Figure. 6. Heme and *Mfsd7c* knockout in MCF7 and 293T cells stimulate OCR and thermogenesis. a Western blotting analysis of MFSD7C in parental MCF7 and 293T cells and their respective *Mfsd7c* knockout cells. **b** to **e** Parental and knockout cells were cultured in the presence of either vehicle or 40 μM heme for 1 hour and OCR was measured using a Seahorse XF96e Analyzer. Shown are representative graphs of OCR output of the indicated cells with or without hematin treatment and their responses to oligomycin, FCCP, and rotenone plus antimycin A (**b**), basal OCR (**c**), maximal OCR (**d**), and ECAR (**e**) from two separate experiments with n=10 biologically independent samples. Data are presented as mean value ± standard deviation. Each dot represents one technical replicate. **f** Comparison of thermogenesis between parental and *Mfsd7c* knockout cells by flow cytometry. Parental and knockout cells were incubated with no probe or FPT for 6 hours, and then washed twice. A portion of FPT-treated cells was treated with heme for 1 hour. FPT fluorescence intensity was quantified by flow cytometry. Shown are representative plots from two separate experiments. P-values were calculated using two-way ANOVA (***$p<0.001$).

Supplementary Figure 7.

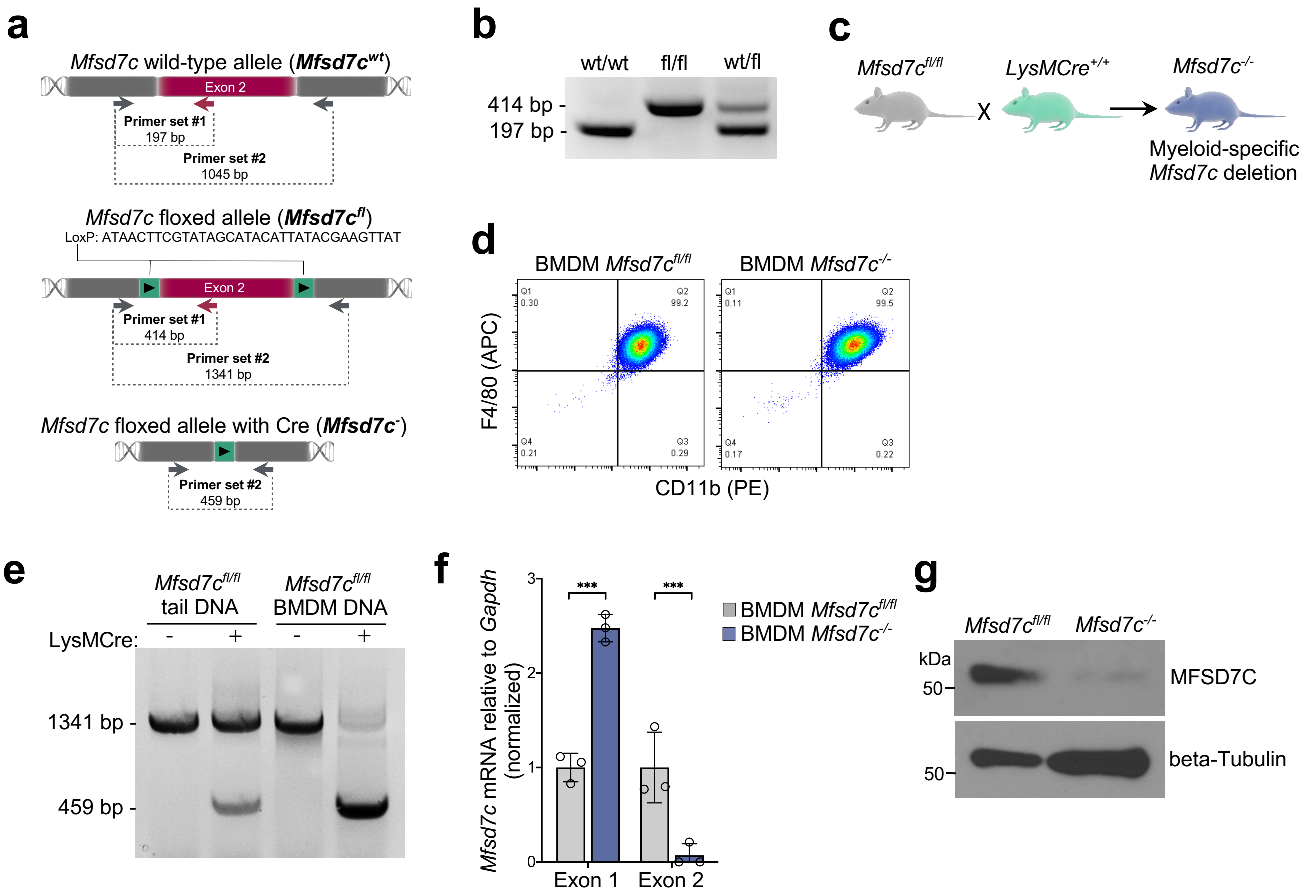

Supplementary Figure. 7. Construction of macrophage-specific *Mfsd7c* knockout mice, characterization and analysis of macrophages. a Schematic representation of wild-type (*Mfsd7c*^{wt}), exon 2-floxed (*Mfsd7c*^{fl}), and exon 2-deleted (*Mfsd7c*$^{-}$) alleles. Primer set #1 and #2 and their respective PCR products used to distinguish between different alleles are shown. **b** PCR analysis of DNA from tails of wild-type (*Mfsd7c*$^{wt/wt}$), floxed *(Mfsd7c*$^{fl/fl}$) and heterozygous (*Mfsd7c*$^{wt/fl}$) mice using primer set #1. Shown are representative data from ten separate experiments. **c** Schematic diagram showing construction of myeloid-specific *Mfsd7c* knockout mice. **d** Comparison of F4/80 and CD11b expression by *Mfsd7c*$^{fl/fl}$ and *Mfsd7c*$^{-/-}$ bone marrow-derived macrophages (BMDM) by flow cytometry gating on live cells. **e** PCR analysis of genomic DNA isolated from tails and BMDM from floxed (*Mfsd7c*$^{fl/fl}$) mice with and without LysM-Cre using primer set #2. Shown are representative data from five separate experiments. **f** Quantitative RT-PCR analysis of *Mfsd7c* exons 1 and 2 with RNA isolated from *Mfsd7c*$^{fl/fl}$ and *Mfsd7c*$^{-/-}$ BMDM. Data are presented as mean value ± standard deviation (n=3 biologically independent samples). P-values were calculated using unpaired t-test (***p<0.001). **g** Western blot analysis of *Mfsd7c*$^{fl/fl}$ and *Mfsd7c*$^{-/-}$ BMDM lysates using antibodies against the C-terminus of MFSD7C and β-tubulin. Shown are representative data from three separate experiments.

Supplementary Figure 8.

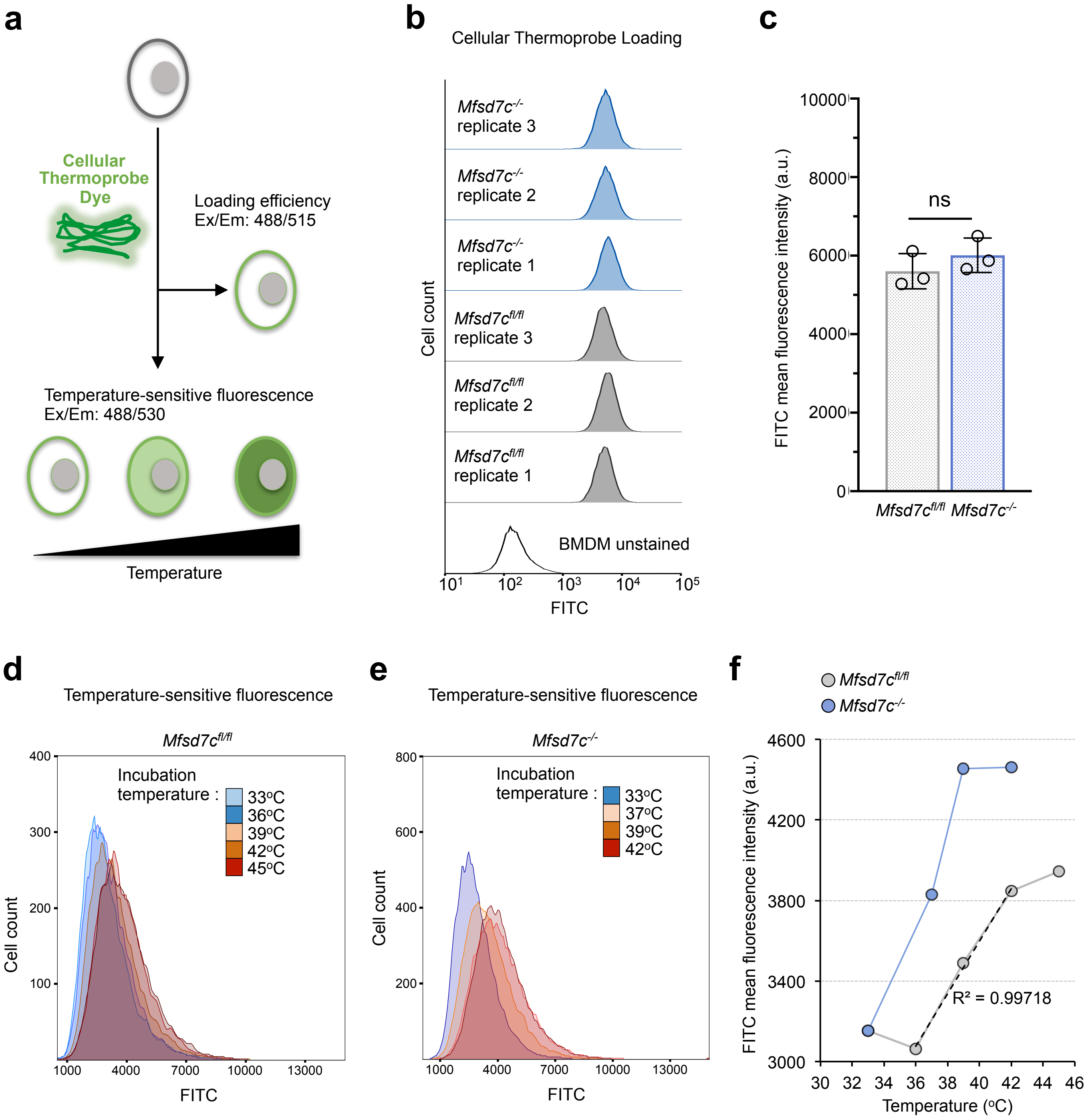

Supplementary Figure 8. Measurement of cellular temperature in mouse BMDM. **a** Schematic representation of the protocol used to analyze dye loading efficiency into BMDM and generation of the cellular temperature standard curve using the temperature-sensitive fluorescent properties of the thermoprobe dye. **b** and **c** Representative thermoprobe dye loading efficiency into *Mfsd7c*$^{fl/fl}$ and *Mfsd7c*$^{-/-}$ BMDM as analyzed by flow cytometry (**b**) and average of three biological replicates (**c**, error bars represent standard deviation). P-values were calculated using unpaired t-test. **d** and **e** Comparison of thermoprobe fluorescence intensities in *Mfsd7c*$^{fl/fl}$ (**d**) and *Mfsd7c*$^{-/-}$ (**e**) BMDM at indicated incubation temperatures by flow cytometry. **f** Plot of incubation temperature versus mean fluorescence intensities (MFI) of thermoprobe loaded into *Mfsd7c*$^{fl/fl}$ and *Mfsd7c*$^{-/-}$ BMDM. Linear trendline (black dashed line) was used to convert MFI to relative cellular temperature of BMDM as shown in Figure 3f (showing one out of three independent biological replicates).

Supplementary Figure 9.

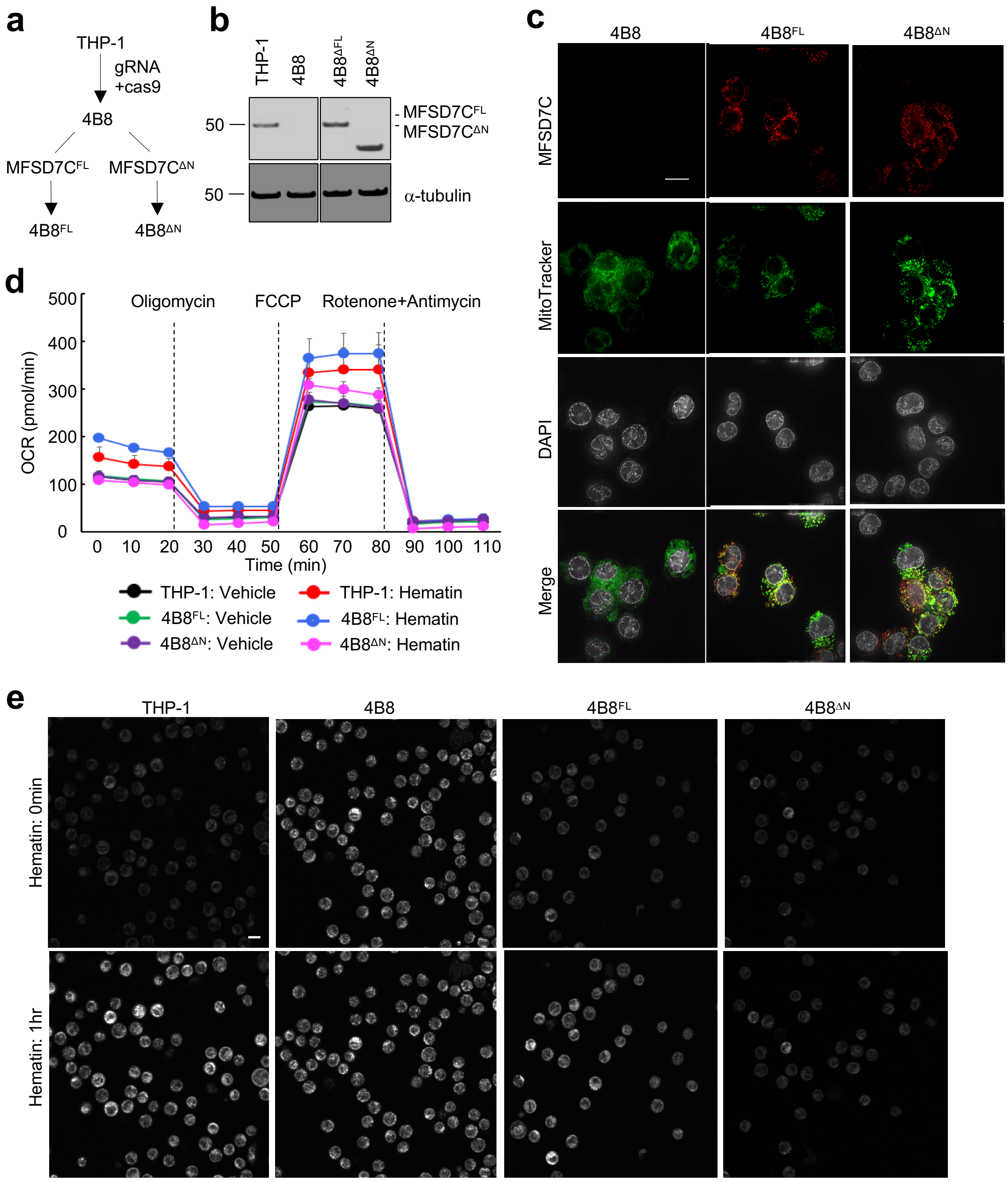

Supplementary Figure. 9. Complementation of 7CKO cells with MFSD7C^{FL} or MFSD7C$^{\Delta N}$. a Scheme showing complementation of 7CKO clone 4B8 with full-length MFSD7C (MFSD7C^{FL}) or N-terminal truncated MFSD7C (MFSD7C$^{\Delta N}$). The complemented 4B8 cells are termed 4B8^{FL} or 4B8$^{\Delta N}$, respectively. **b** Complementation with either MFSD7C^{FL} or MFSD7C$^{\Delta N}$ restores MFSD7C expression. MFSD7C was detected with a polyclonal anti-MFSD7C antibody recognizing the C-terminus of MFSD7C. Shown are representative data from five separate experiments. **c** Localization of MFSD7C^{FL} and MFSD7C$^{\Delta N}$ in mitochondria. Immunofluorescent labelling of MFSD7C (red) and MitoTracker (green) in 4B8, 4B8^{FL} and 4B8$^{\Delta N}$ cells. Co-localization between MFSD7C and MitoTracker appears as yellow on merged images (Merge). Nuclei (gray) were labeled using DAPI. Shown are representative data from three separate experiments. **d** Representative graphs of OCR outputs from the XF96 analyzer of THP-1, 4B8^{FL} and 4B8$^{\Delta N}$ cells with or without heme treatment. **e** THP-1, 4B8, 4B8^{FL} and 4B8$^{\Delta N}$ cells were incubated with FPT for 6 hours, and then washed and reseeded in 35 mm glass bottom dish. Cells in the same field were imaged before and after hematin treatment. Shown are representative data from three separate experiments. Fluorescent intensity was quantified and shown in Figure 4d.

Supplementary Figure 10.

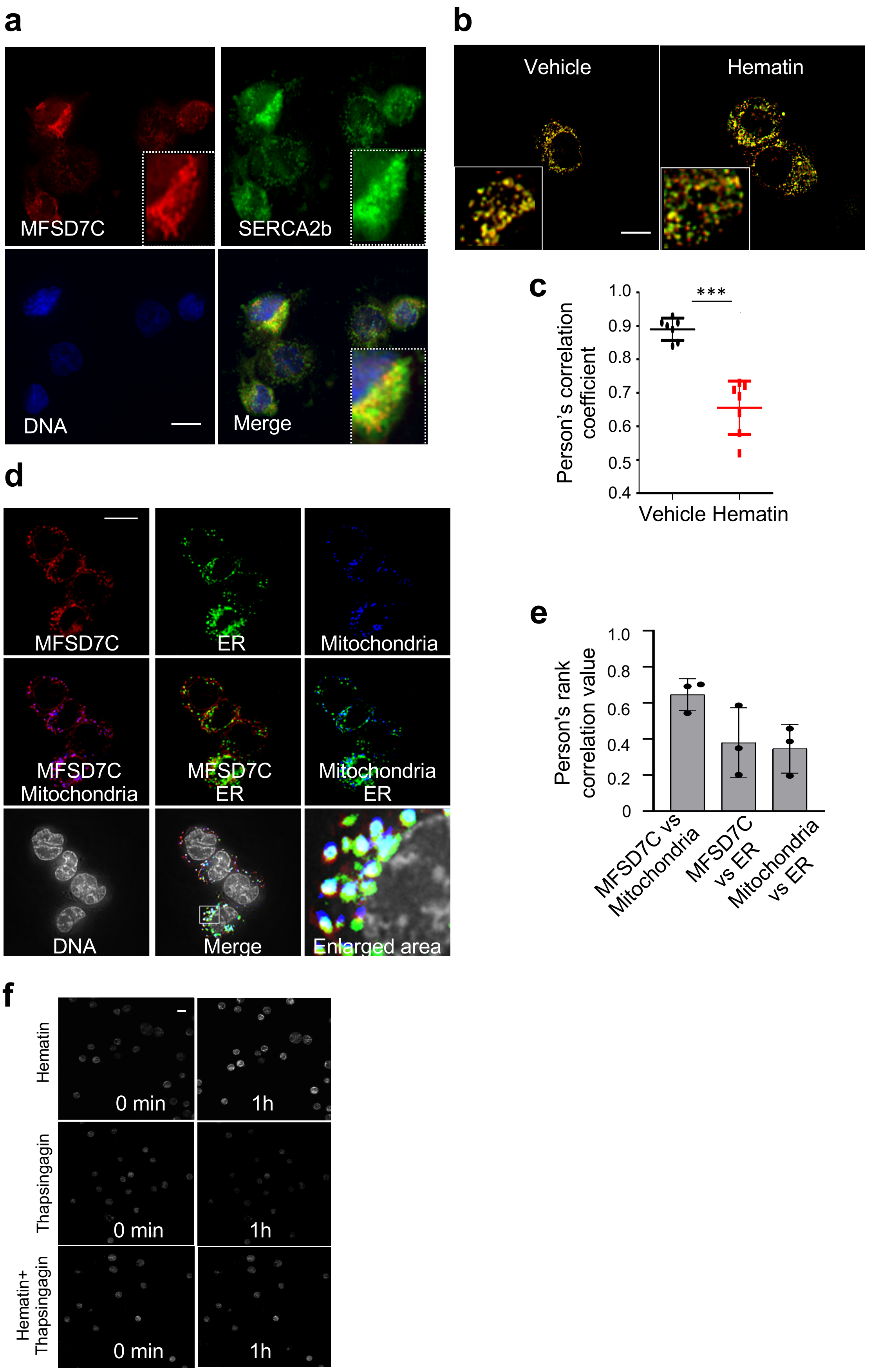

Supplementary Figure 10. Co-localization of MFSD7C and SERCA2b. a Confocal analysis of MFSD7C and SERCA2b co-localization in THP-1 cells. Shown are representative images of THP-1 cells stained for MFSD7C (red), SERCA2b (green), nuclei (blue) and merged image. **b** and **c** Heme disrupts MFSD7C-SERCA2b co-localization. THP-1 cells were not treated or treated with 40 μM of heme for 1 hour and then stained for MFSD7C, SERCA2b and nuclei as in (**a**). Co-localization between MFSD7C and SERCA2b was quantified. Shown are representative merged images (**b**) and comparison of Pearson's correlation coefficients values with or without heme treatment (**c**) (in **c**, n=7 biologically independent samples). P-values were calculated using unpaired t-test (***p<0.05). **d** and **e** MFSD7C localizes at the ER-mitochondrial junction. THP-1 cells were stained for MFSD7C (red), ER (green), mitochondria (blue), and DNA (grey). Shown are representative single and merged images (**d**). Enlarged image of the boxed area is shown in the last panel (bottom-right). Pearson's rank correlation values between the indicated comparisons are shown (**e**). (n=3 biologically independent samples). **f** Thapsigargin inhibits heme-induced thermogenesis in THP-1 cells. THP-1 cells were incubated with FPT for 6 hours, washed, treated with or without 4 μM thapsingargin for 2 hours. The cells were washed and reseeded in poly-lysine coated glass bottom dishes. After cells attached to the glass, medium containing 40 μM heme was added. Cells in the same field were imaged immediately (0 min) and again 1 hour later. Representative images from one of two experiments are shown. Data in (**c**) and (**d**) are presented as mean values ± standard deviation. Scale bar: 10 μm.

Supplementary Table 1. Primers used in this study.

Figure	Name	Forward (5'-3')	Reverse (5'-3')
S6	Set #1	gaactgtgtatcagtcaagttgtcaagg	gagctcattggccagccagc
S6	Set #2	gaactgtgtatcagtcaagttgtcaagg	gacagggtagtagtctggctgc
S6	LysM-Cre	cccagaaatgccagattacg	cttgggctgccagaatttctc
S6	*Mfsd7c* Exon1 qPCR	cagcgtgatcaaggtgagcaag	attgtgaccgggaagaggtgag
S6	*Mfsd7c* Exon2 qPCR	aagcttgcctaccacatcag	gcttgggcttctccttgaata
S2b	BglII-NHEI-GFP-F/ BamHI-2A-GFP-R	gaattcAGATCTGCTAGCATGG TGAGCAAGGGCGAGG	GGTGGATCCAGGTCCAGG GTTCTCCTCCACGTCTCCA GCCTGCTTCAGCAGGCTG AAGTTAGTAGCTCCGCTTC CCTTGTACAGCTCGTCCAT GCC
S2b	Mfsd7c-SgfI-F/ AC-Myc-DDK-KpnI-R	cggaattcgcgatcgcCATGGTGAA TGAAAGTCTCAAC	cggaattc*ggtacc*gtttaaaccttatc gtcgtcatcc
S3b	BamHI-P2A-mCherry-F/ mCherry-WRPE-R	CGATAAGGGATCCGGCGCAA CAAACTTCTCTCTGCTGAAAC AAGCCGGAGATGTCGAAGAG AATCCTGGACCGATGGTGAG CAAGGGCGAGGA	TGATTGTCGACTTAACGCG TTCACTTGTACAGCTCGTC CATGC
S3b	mCherry-WRPE-F/ PmeI-R	GCATGGACGAGCTGTACAAG TGAACGCGTTAAGTCGACAA TCA	tcgaggctgatcagcgggtt
S3b	BamHI-P2A-F/ PmeI-R	CGATAAGGGATCCGGCGCAA	tcgaggctgatcagcgggtt
S3b	gRNA-1-Forward/ gRNA-1-Reverse	caccgCTCGTCCCGGTCTTCAA TGT	aaacACATTGAAGACCGGG ACGAGc
S3b	gRNA-2-Forward/ gRNA-2-Reverse	caccgCACCATGCGATTCAGAA GAG	aaacCTCTTCTGAATCGCAT GGTGc
S3b	gRNA-3-Forward/ gRNA-3-Reverse	caccgCTATAGCTTGGAATTGC GAT	aaacATCGCAATTCCAAGCT ATAGc
S3b	F1/R1 for genomic test of MFSD7c KO-1	CCAGATATGGGAGTAGAGGA	ACAAAGTGCATAGGACCA GC
S3b	F2/R2 for genomic test of MFSD7c KO-2	TGTCCAGGACTTCTACTGAC	GCTCAGAAACACTAACTAG C
S3b	F3/R3 for genomic test of MFSD7c KO-3	CTGGGTGACAGAGCGAGACT	ACCAACAGGCATTTGTCA GA

Figure	Name	Forward (5'-3')	Reverse (5'-3')
S2c	mu-GAPDH-qPCR-F/ mu-GAPDH-qPCR-R	CAAGAAGGTGGTGAAGCAGG	TTGTCATTGAGAGCAATGCC
S2c	mu-Mfsd7c-qPCR-F/ mu-Mfsd7c-qPCR-R	TGCCTTAGCGACCACTGATGCT	GGATAAGGCGTAGAAAGCACCAG
4a-f	MFSD7C-AsiSI-F/MFSD7C-KpnI-R	GAGAACCCTGGACCTGCGATCGCCATGGTGAATGAAGGTCCCAA	gCGGTCcTCgATaTTaGGTACCAAAAC
4a-f	MFSD7C-KpnI-F/ MFSD7C-XmaI-R	tAAtATcGAgGACCGcGACGAGCTTG	cCGgTTCAGgAGgGTaGACAAGGCAT
4a-f	MFSD7C-XmaI-F/ MFSD7C-MluI-R	tACcCTcCTGAAcCGgATGGTGATCT	CTGCTCGAGCGGCCGCGTACGCGTGAGATGATCCTCTGACACAG
4a-f	ΔNTD-MFSD7C-AsiSI-F/MFSD7C-KpnI-R	GAGAACCCTGGACCTGCGATCGCCCGTTGGGCCGTGGTCCTGGT	CGGTCcTCgATaTTaGGTACCAAAAC

Supplementary Table 2. Plasmids used in this study.

Figure	Name	Description	Originator
1, S1	GST-His-tag-SUMO-NTD	NTD expression in E.coli	N.A.I
1, S1	GST-His-tag-SUMO-Mutant NTD	Mutant NTD expression in E.coli	N.A.I
1d	pCMV6-AC-DDK-HMOX1	HMOX1 expression in mammalian cells	Origene MR203944
1d	pCMV6-AC-DDK-Cyc1	Cyc1 expression in mammalian cells	Origene MR204721
1d	pCMV6-AC-DDK-Cox4i1	Cox4i1 expression in mammalian cells	Origene MR218332
1d	pCMV6-AC-DDK-Ndufa4	Ndufa4 expression in mammalian cells	Origene MR216909
1d	pCMV6-AC-DDK-ATP5c1	ATP5c1 expression in mammalian cells	Origene MR204152
1d	pCMV6-AC-DDK-ATP5h	ATP5h expression in mammalian cells	Origene MR201260
1d	pCMV6-AN-3HA	Empty backbone	Origene PS100066
1d and S2b	pCMV6-AC-DDK-MFSD7c	MFSD7c expression in mammalian cells	Origene MR208748
1d	pCMV6-AN-3HA-MFSD7C	MFSD7c expression in mammalian cells	Y.L
4a-f	pLKO.1-GFP-P2A-FL-MFSD7C-Myc-DDK	MFSD7c expression in mammalian cells	Y.L and T.D
4a-f	pLKO.1-GFP-P2A-ΔN-MFSD7C-Myc-DDK	MFSD7c expression in mammalian cells	Y.L and T.D
S2e	pCMV6-AC-GFP	Empty backbone	Origene PS100010
S2e	pCMV6-AC-GFP-MFSD7C	MFSD7c expression in mammalian cells	Y.L
S2b	pLKO.1 GFP shRNA	shRNA backbone	Addgene 30323
S2b	pLKO.1-GFP-P2A-MFSD7C-Myc-DDK	MFSD7c expression in mammalian cells	Y.L
S3b-e	LentiCRISPR v2	CRISPR backbone	Addgene 52961
S3b-e	pLentiCRISPR-v2-mCherry-hu7CKO-1	Knockout MFSD7c in mammalian cells	Y.L
S3b-e	pLentiCRISPR-v2-mCherry-hu7CKO-2	Knockout MFSD7c in mammalian cells	Y.L

Figure	Name	Description	Originator
S3b-e	pLentiCRISPR-v2-mCherry-hu7CKO-3	Knockout MFSD7c in mammalian cells	Y.L
