# Supplementary material for: MFSD7C switches mitochondrial ATP synthesis to thermogenesis in response to heme
Source: Nat Commun. 2020 Sep 24;11:4837. doi: 10.1038/s41467-020-18607-1 (PMC7515921; doi:10.1038/s41467-020-18607-1)
Supplement: Supplementary file 2 — Peer Review File [file 41467_2020_18607_MOESM2_ESM.docx]

Peer Review Information

**Journal:** Nature Communications

**Manuscript title:** MFSD7C Switches Mitochondrial ATP Synthesis to Thermogenesis in Response to Heme

**Corresponding author name(s):** Prof Jianzhu Chen

**Editorial notes:**

**Transferred manuscripts**

This manuscript has been previously reviewed at another journal that is not operating a transparent peer review scheme. This document only contains reviewer comments and rebuttal letters for versions considered at *Nature Communications*.

**Reviewer comments & decisions:**

**Reviewer comments, first version:**

Reviewer #1 (Remarks to the Author):

In this paper, the authors describe a new mechanism that allow uncoupling of mitochondrial respiration to switch ATP synthesis to thermogenesis. They showed that the MFS transporter MFSD7C binds heme through its N-terminal domain, and co-immunoprecipitate with several components of the ETC complexes and with SERCA 2b. Data indicate that when heme levels are low, MSFD7C promotes ATP synthesis and inhibits thermogenesis. According the model proposed by the authors when heme concentration is increased, the binding of heme to MFSD7C results in reduced cell respiration and stabilization of SERCA 2b that promotes thermogenesis.
The model is interesting but not adequately supported by experimental data. My major concern is about the fact that most data were obtained with overexpressed proteins and in non-physiological experimental conditions (10-40 µM heme mimics a condition of heme overload associated to hemolysis, not a physiologic situation).
Data on mitochondrial localization of MFSD7C must be improved. Has MFSD7C a mitochondrial targeting sequence? Usually this sequence is at the N-term of the protein. The authors stated that the N-tem truncated protein maintains the mitochondrial localization. This is unexpected. A cell fractionation is required. Moreover, data on the endogenous protein need a control of antibody specificity: the authors have to include as a negative control MFSD7C KO cells.
Co-immunoprecipitation with components of ETC and SERCA 2b must be confirmed with the endogenous proteins.
It is very hard to imagine that a protein localized in the inner mitochondrial membrane (not demonstrated) may interact with a ER protein. A precise localization of the endogenous proteins might elucidate this point.
In Fig. 2b-d, it is not clear which differences are significant.


Reviewer #2 (Remarks to the Author):

In the manuscript entitled “MFSD7C Switches Mitochondrial ATP Synthesis to Thermogenesis in Response to Heme”, the authors provided evidences showing the solute carrier protein MFSD7C plays a role in modulating mitochondrial ATP synthesis and thermogenesis. The study was very well executed and the manuscript was nicely prepared. There is not much to be criticized on the paper itself except that in Fig. S6b, the inclusion of 4B8WT is confusing, what is the difference between 4B8 and 4B8WT given the WT MFSD7C could also be targeted by the CRISPR-Cas9 system? Where was 4B8WT cell line used in the subsequent experiments? It was not described in any results thereafter. However, the outmost concern on this study lies in its physiological relevance. To my knowledge, in mammals, the major thermogenic sites are brown fat, and to a lesser extent beige fat that resides within the white fat depot. There were also reports suggesting thermogenic activity in skeletal muscle. But this report suggests that human monocytic cells are also thermogenic. What is the physiological evidence for that? In order to establish such a model the authors need to test their idea in monocytic cell-specific Mfsd7c KO animals. Moreover, is there evidence showing food intake would affect Heme levels in blood which in turn modulates thermogenesis?


Reviewer #3 (Remarks to the Author):

The manuscript by Li and colleagues reports on a new mechanism for thermogenesis, that can be regulated by heme binding to MFSD7C (aka FLVCR2). This is an interesting hypothesis and the in vitro data supporting heme binding to MFSD7C, and the interaction of MFSD7C to components of the ETC are in the most case strong. Likewise, the evidence that heme increases mitochondrial respiration and heat production is persuasive, although the use of the fluorescent polymeric thermometer requires further validation/explanation. The weakest part of the study concerns the mechanism by which MFSD7C increases thermogenesis and the (patho)physiological relevance of this pathway.

Major Points
1. The use of the fluorescent polymeric thermometer (FTP) reporter requires further explanation/validation. This would require more details of the synthesis of the reporter (details of the compound used). It is not clear to this reviewer how the fluorescent intensity can be used as a direct measure of temperature. Wouldn’t this require FLIM measurement (i.e. how can the uptake of the dye be assumed to be the same in the different cell lines)?

2. The authors present extensive evidence for the interaction of MFSD7C with various proteins, but this is always determined using in vitro approaches. Can the authors provide evidence for these interactions in endogenous conditions (i.e. in mammalian tissues)?

3. Related to the above point, the authors use primarily HEH293T and THP-1 cells, but what cell types/tissues do the authors imagine this mechanism might operate in? Do the authors have any evidence that the interaction is specific for SERCA2b? Other studies have reported that SERCA1a and SERCA2a are involved in futile calcium cycling and thermogenesis is skeletal muscle, whilst SERCA2b may be the predominant isoform responsible for calcium cycling in beige adipocytes. An important point in the current study would be to distinguish which isoforms are the most physiologically relevant (which in turn would probably reveal which tissues/cell types are important for this mechanism). In muscle, sarcolipin plays an important role in thermogenesis. Is there any evidence for a role of sarcolipin in the current study? Understanding these issues would require better characterisation of the endogenous levels of MFSD7C and components of the calcium cycling pathway (SERCA and the ryanodine receptors), rather than using in vitro cell models.

4. Why is the ATP to ADP ratio so low (Fig. S4b)? ATP levels would be expected to be 5-10 fold higher than ADP levels.

**Author rebuttal, first version:**

**Reviewer #1 (Remarks to the Author):**

In this paper, the authors describe a new mechanism that allow uncoupling of mitochondrial respiration to switch ATP synthesis to thermogenesis. They showed that the MFS

transporter MFSD7C binds heme through its N-terminal domain, and co-immunoprecipitate with several components of the ETC complexes and with SERCA 2b. Data indicate that when heme levels are low, MSFD7C promotes ATP synthesis and inhibits thermogenesis. According the model proposed by the authors when heme concentration is increased, the binding of heme to MFSD7C results in reduced cell respiration and stabilization of SERCA 2b that promotes thermogenesis.

**Response:**

We would like to note that according to our proposed model, binding of heme to MFSD7C results in *enhanced* cellular respiration and stabilization of SERCA2b that promotes thermogenesis.

1. The model is interesting but not adequately supported by experimental data. My major concern is about the fact that most data were obtained with overexpressed proteins and in non-physiological experimental conditions (10-40 µM heme mimics a condition of heme overload associated to hemolysis, not a physiologic situation).

**Response:**

In the following response, we address the non-physiological concentration of heme. We

address the issue with protein overexpression in response to point #3.

The labile (non-protein-bound) heme concentration was estimated to be 614 ± 214 nM in lung fibroblast cell extract (Masuda and Takahashi, 2006) and 433 ± 125 nM in the cytosol of HEK293 cells (Yuan et al 2016). The cytosolic labile heme concentrations in general are thought to be less than 1 µM, given that the *Km* of heme oxygenase-1 is ∼1 µM (Sassa, 2004; Yuan et al 2016). In erythrocytes, labile heme concentration was estimated to be around 20 µM (Aich et al, 2015).

As far as we are aware, there is no reliable data available on mitochondrial matrix labile heme concentration. But based on cytosolic and erythrocyte data a reasonable estimate is 1-20 µM because heme is synthesized in mitochondrial matrix, and demand for heme is higher in mitochondria than in the cytosol.

In human serum, total heme concentration (free and protein-bound) is ~15 µM in

healthy subjects and 2-10 times higher in sickle cell disease patients (Oh et al 2016). However, heme scavengers such as hemopexin, albumin, and lipoproteins bind to labile heme very tightly (hemopexin KD is in low nM to pM range) and under normal conditions there is very little labile heme in the blood.

Because of the strict control of free heme in the serum, extracellular liable heme does not usually reach 10-40 µM under physiological conditions. However, MFSD7C is a mitochondrial protein where it likely experiences higher heme concentrations. As shown in response to point #3, we have now shown that adding 5 µM heme into cell culture is sufficient to modulate interactions between endogenous MFSD7C and ATP5h, SERCA2b and Hmox1. Furthermore, in our co-IP experiment, heme was added to cell cultures in the presence of serum. Serum albumin and other proteins likely bind and absorb some of the added heme. Therefore, the actual heme level in the cell and mitochondria is likely less than 5-40 µM. Based on these considerations, we believe that 5 to 40 µM of heme in our

1

experiments are relevant concentrations. These heme concentrations are certainly relevant under pathological conditions, such as hemolysis, where the local liable heme concentrations could be much higher.

In the revised manuscript, we have discussed the relevance of heme concentrations used in relation to physiological and pathological conditions in the Discussion.

Masuda T, Takahashi S. Chemiluminescent-based method for heme determination by reconstitution with horseradish peroxidase apo-enzyme. *Anal Biochem.* 2006; 335:307-9.

Yuan X, Rietzschel N, Kwon H, et al. Regulation of intracellular heme trafficking revealed by subcellular reporters. *Proc Natl Acad Sci U S A*. 2016; 113(35): E5144–E5152.

Sassa S*.* Why heme needs to be degraded to iron, biliverdin IXalpha, and carbon monoxide? *Antioxid Redox Signal.* 2004; 6*(*5*):*819*–*824*.*

Aich A, Freundlich M, Vekilov PG. The free heme concentration in healthy human erythrocytes. *Blood Cells Mol Dis*. 2015; 55(4):402–409.

Oh JY, Hamm J, Xu X, et al. Absorbance and redox based approaches for measuring free heme and free hemoglobin in biological matrices. *Redox Biol*. 2016; 9:167–177.

2. Data on mitochondrial localization of MFSD7C must be improved. Has MFSD7C a mitochondrial targeting sequence? Usually this sequence is at the N-term of the protein. The authors stated that the N-tem truncated protein maintains the mitochondrial localization. This is unexpected. A cell fractionation is required. Moreover, data on the endogenous protein need a control of antibody specificity: the authors have to include as a negative control MFSD7C KO cells.

**Response:**

According to several mitochondrial localization prediction softwares (DeepMito, SignalP-5.0, MitoFates), MFSD7C does not contain a mitochondrial targeting pre-sequence. In addition, MFSD7C is not listed in the MitoCarta inventory of mitochondrial proteins. One possibility is that like other mitochondrial metabolite carriers, MFSD7C is imported into mitochondria via the carrier import pathway that does not require a cleavable pre-sequence, but uses other internal targeting elements (Wiedemann and Pfanner, 2017). In agreement with this possibility, MFSD7C co-immunoprecipitated with TIM50 and IMMT (MIC60) (Fig. 1d), two proteins involved with mitochondrial import machinery. This could explain why MFSD7C N-terminal domain deletion mutant retains mitochondrial localization.

Nevertheless, per reviewer’s suggestion, we have performed cell fractionation followed by Western blotting to further localize endogenous MFSD7C. We used whole mouse brain extract, where MFSD7C expression is the highest (Brasier et al 2004). As shown in Fig. 1g of the revised manuscript (left), the majority of MFSD7C localizes to the mitochondrial fraction (10,000g precipitate), similar to known mitochondrial proteins VDAC and COX4I1. In contrast, known cytoplasmic proteins GAPDH and nucleophosphamin 1 (NPM1), endoplasmic reticular (ER) protein calreticulin (CalR) and SERCA2b, and autophagosomal protein LC3 are predominantly present in the supernatant fraction. These results further support mitochondrial localization of MFSD7C. The results have been added to the revised manuscript as Fig. 1g.

2

**Fig. 1g**: Localization of endogenous MFSD7C in mitochondria by cell fractionation. Mouse whole brain extract was fractionated using differential centrifugation to enrich for mitochondria and analyzed by immunoblotting against indicated proteins. WCE: whole cell extract, Sup: supernatant, Mito: crude mitochondria fraction.

To address antibody specificity using MFSD7C KO cells, we have used MFSD7C KO cells in co-IP experiments (see response to point #3 below). In addition, we have performed immunofluorescence staining using the polyclonal antibody specific for the C-terminal domain of MFSD7C. As shown in Supplementary Fig. 2d of the revised manuscript (below), the polyclonal antibody stained parental THP1 cells but not 7CKO THP1 cells (clone 3D12). Consistently, the polyclonal antibody stained 7CKO cells that expressed MFSD7C as a transgene. These results are included as Supplementary Fig. 2d of the revised manuscript.

**Supplementary Fig. 2d**. Specificity of the polyclonal antibody against the C-terminal domain of MFSD7C. Parental THP-1 cells, clone 3D12 of 7CKO in THP-1 cells, and 3D12 cells transduced with a full length of MFSD7C in lentiviral vector (3D12R) were stained with polyclonal antibody specific for the C-terminal domain of MFSD7C and DAPI. Shown are images of endogenous MFSD7C (top row) and merged images with DAPI (bottom row) in parental THP-1 cells (left column), 3D12 clone (middle column), and 3D12R (right column). Scale bar, 10 µm.

Savojardo C, Bruciaferri N, Tartari G, Martelli PL, Casadio R. DeepMito: accurate prediction of protein sub-mitochondrial localization using convolutional neural networks. Bioinformatics. 2020;36(1):56–64. doi:10.1093/bioinformatics/btz512

Almagro Armenteros JJ, Tsirigos KD, Sønderby CK, et al. SignalP 5.0 improves signal peptide predictions using deep neural networks. Nat Biotechnol. 2019;37(4):420–423. doi:10.1038/s41587-019-0036-z

Fukasawa Y, Tsuji J, Fu SC, Tomii K, Horton P, Imai K. MitoFates: improved prediction of mitochondrial targeting sequences and their cleavage sites. Mol Cell Proteomics. 2015;14(4):1113–1126. doi:10.1074/mcp.M114.043083

Calvo SE, Clauser KR, Mootha VK. MitoCarta2.0: an updated inventory of mammalian mitochondrial proteins. Nucleic Acids Res. 2016;44(D1):D1251–D1257. doi:10.1093/nar/gkv1003

Wiedemann, N. and Pfanner, N. Mitochondrial Machineries for Protein Import and Assembly. *Annu. Rev. Biochem.* 2017. 86:685–714

Brasier G, Tikellis C, Xuereb L, Craigie J, Casley D, Kovacs CS, Fudge NJ, Kalnins R, Cooper ME, Wookey PJ. Novel hexad repeats conserved in a putative transporter with restricted expression in cell types associated with growth, calcium exchange and homeostasis. Exp Cell Res. 2004 Feb 1;293(1):31-42.

3

3. Co-immunoprecipitation with components of ETC and SERCA 2b must be confirmed with the endogenous proteins.

**Response:**

Per reviewer’s suggestion, we have performed co-immunoprecipitation of endogenous MFSD7C, ATP5h, SERCA2b and Hmox1 using bone marrow-derived macrophages from C57BL/6 mice with exon 2 of *Mfsd7c* flanked with loxP sites (*Mfsd7c^fl/fl^*) or deleted (*Mfsd7c^-/-^*) specifically in macrophages (see revised Supplementary Materials for details). As shown in Fig. 1f of the revised manuscript (below), the endogenous MFSD7C co-immunoprecipitated with endogenous ATP5h, SERCA2b and Hmox1 in wildtype (*Mfsd7c^fl/fl^*) macrophages but not in *Mfsd7c^-/-^* macrophages. In addition, the co-IP was sensitive to heme treatment as in THP-1 cells: i.e., co-IP of MFSD7C with ATP5h and SERCA2b was reduced by increasing concentrations of heme, while co-IP of MFSD7C with Hmox1 was enhanced by increasing concentrations of heme. Thus, interactions between endogenous MFSD7C and ATP5h, SERCA2b and Hmox1 also occur in primary macrophages and are sensitive to heme. We have added these results in the revised manuscript as Fig. 1f.

|  | **Fig. 1f**: Co-immunoprecipitation of endogenous MFSD7C with ATP5h, SERCA2b and Hmox1 in bone marrow-derived macrophages. The Co-IP experiments were performed using PierceTM Classic IP Kit following the manufacturer’s protocol (Thermo Fisher, #26146). Briefly, 1 mg of protein extracts of bone marrow-derived macrophages from either *Mfsd7c^fl/fl^* or *Mfsd7c^-/-^*mice were incubated with 10 µg of primary anti-SERCA2b (CST, #4388), anti-ATP5h (ab241164), anti-Hmox1 (CST, 70081) antibodies, and then immunoprecipitated with bead-bound secondary anti-IgG antibodies. Negative controls were performed with the same amount of protein extract and immunoprecipitated with the anti-IgG antibodies in the absence of primary antibodies. Immunoprecipitates were resolved by SDS–PAGE, transferred onto nitrocellulose membrane, and blotted as indicated. Shown are immunoblotting of MFSD7C, ATP5h, SERCA2b, and Hmox1 on whole cell lysates or immunoblotting of MFSD7C on anti-ATP5h, anti-SERCA2b and anti-Homx1 immunoprecipitates. |
| --- | --- |

4. It is very hard to imagine that a protein localized in the inner mitochondrial membrane (not demonstrated) may interact with a ER protein. A precise localization of the endogenous proteins might elucidate this point.

**Response:**

Contacts between endoplasmic reticulum (ER) and mitochondria have been established for several decades and these contacts are involved in diverse functions, from ion and lipid transfer, to signaling, and to membrane dynamics (Csordas et al, 2018). Studies have identified many protein complexes that mediate the interactions between ER and mitochondria. For example, IP3R-GRP75-VDAC1 complex might interact with MCU in Ca^++^ transfer, where MCU resides on the inner mitochondrial membrane whereas IP3R resides on ER membrane, and GRP75 and VDAC1 reside on the outer mitochondrial membrane (Arruda and Hotamisligil, 2015). Similarly, ER membrane complex (EMC) may interact with OPA1 and MICOS on the inner mitochondrial membrane through SLC25A46 on the outer mitochondrial membrane (Csordas et al 2018). In particular, SERCA2b on the ER was

4

shown to interact with HAX-1 in mitochondria (Vafiadaki et al 2009), although whether HAX-1 resides on the outer or inner mitochondrial membrane is unknown.

In our studies, we show that MFSD7C and SERCA2b co-immunoprecipitate in both overexpression (Fig. 1e) and endogenous setting (see response to point #3 above). Both MFSD7C and SERCA2b reside in the ER-mitochondrial contact points by immunofluorescence and confocal microscopy (Supplementary Fig. 9). However, we must stress that these results do not mean direct physical interaction between SERCA2b and MFSD7C. Most likely, both SERCA2b and MFSD7C reside in the same protein complex, similar to other protein complexes described between ER and mitochondria. Another possibility is that MFSD7C resides on the outer mitochondrial membrane, enabling it to interact with SERCA2b on the ER and ETC complexes on the inner mitochondrial membrane. To resolve these possibilities, precise localization of MFSD7C by immune-labeling plus electron microscopy or using other approaches is required, but is challenged by a lack of monoclonal anti-MFSD7C antibody at the present time.

We have revised the Discussion to consider the possibility that MFSD7C resides on the outer mitochondrial membrane as well. The two scenarios are depicted in Fig. 5g-h of the revised manuscript (below).

**g	h**

**Fig. 5g,h**. Proposed models of MFSD7C regulation of mitochondrial respiration in response to heme, with MFSD7C residing in the inner (**g**) or outer (**h**) mitochondrial membrane. When heme level is low, the interactions result in SERCA2b ubiquitination and degradation, leading to coupled mitochondrial respiration: increased ATP synthesis and reduced thermogenesis. When heme level is high, heme binding to the NTD of MFSD7C disrupts its interactions with ETC components and SERCA2b, leading to SERCA2b stabilization and uncoupled mitochondrial respiration: increased thermogenesis and reduced ATP synthesis.

Csordas G, Weaver D, and Hajnoczky G. Endoplasmic reticular-mitochondrial contactology: Structure and signaling function. Trends Cell Biol. 2018, 28:523-540.

Arruda, A. P., & Hotamisligil, G. S. Calcium homeostasis and organelle function in the pathogenesis of obesity and diabetes. Cell Metabolism, 2015, 22,381–397.

Vafiadaki E, Arvanitis D A, Pagakis S N, et al. The anti-apoptotic protein HAX-1 interacts with SERCA2 and regulates its protein levels to promote cell survival. J Mol Biol Cell. 2009, 20:306-318.

5

5. In Fig. 2b-d, it is not clear which differences are significant.

**Response:**

We have performed two-way ANNOVA analyses and added the significant differences in Fig. 2b-d of the revised manuscript. Similarly, we have added additional significant differences in Fig. 4a-d.

**Reviewer #2 (Remarks to the Author):**

In the manuscript entitled “MFSD7C Switches Mitochondrial ATP Synthesis to Thermogenesis in Response to Heme”, the authors provided evidences showing the solute carrier protein MFSD7C plays a role in modulating mitochondrial ATP synthesis and thermogenesis. The study was very well executed and the manuscript was nicely prepared.

1. There is not much to be criticized on the paper itself except that in Fig. S6b, the inclusion of 4B8WT is confusing, what is the difference between 4B8 and 4B8WT given the WT MFSD7C could also be targeted by the CRISPR-Cas9 system? Where was 4B8WT cell line used in the subsequent experiments? It was not described in any results thereafter.

**Response:**

Although we described 4B8^WT^ in the figure legend, we agree with the reviewer that inclusion

of 4B8^WT^ is confusing. Therefore, we have removed it from the revised manuscript.

2. However, the outmost concern on this study lies in its physiological relevance. To my knowledge, in mammals, the major thermogenic sites are brown fat, and to a lesser extent beige fat that resides within the white fat depot. There were also reports suggesting thermogenic activity in skeletal muscle. But this report suggests that human monocytic cells are also thermogenic. What is the physiological evidence for that? In order to establish such a model the authors need to test their idea in monocytic cell-specific Mfsd7c KO animals.

**Response:**

Yes, the reviewer is correct that brown and beige adipose tissues and muscles are major thermogenic organs for producing heat to maintain stable body temperature in endotherms. Following cold exposure, heat is generated by muscle via shivering thermogenesis and by brown and beige adipose tissues via non-shivering thermogenesis. UCP1 is critical for thermogenesis in the brown adipose tissue while SERCA2b is critical for thermogenesis in the beige adipose tissue. Although these mechanisms of thermogenesis are essential to maintain body temperature in endotherms in response to cold exposure and may play a significant role in maintaining body temperature under ambient temperatures, there are likely other mechanisms of thermogenesis that contribute to the maintenance of the body temperature in endotherms. For example, pigs do not have the *Ucp1* gene and they do not shiver constantly, therefore thermogenesis from the brown adipose tissue and muscle do not contribute to thermogenesis under the ambient temperatures. It is unlikely that pigs maintain the constant body temperature through the beige adipose tissues only.

According to thermodynamics, all chemical reactions dissipate energy and this energy is released in the form of heat. Therefore, every metabolically active cell generates heat. Studies in the 1970s have shown that most of the energy stored in the mitochondrial proton gradient is released as heat instead of driving ATP synthesis, even though many biochemistry textbooks focus solely on ATP synthesis following the oxidation of reduced carbon sources. Based on these considerations, although monocytes are not thermogenic organs they do produce their own heat. Broadly, heat released from each cell in the body likely contributes to maintaining the constant temperature of endotherms.

6

Nevertheless, we have generated myeloid-specific *Mfsd7c* knockout mice. These mice maintain constant body temperature without shivering, suggesting macrophages are not a key thermogenic cell type for maintaining body temperature in mice. However, *Mfsd7c*-deficient macrophages exhibit increased levels of OCR, ECAR, and thermogenesis, but reduced mitochondrial membrane potential, demonstrating thermogenic ability in primary macrophages. We have included these data as Fig. 3 (below) and Supplementary Fig. 6 (below) and 7 of the revised manuscript.

**Fig. 3.** Loss of *Mfsd7c* stimulates OCR, ECAR and thermogenesis in bone marrow-derived macrophages. Macrophages were derived from exon 2 floxed (*Mfsd7c^fl/fl^*) C57BL/6 mice and C57BL/6 mice with exon 2 deletion in macrophages (*Mfsd7c*^-/-^). Representative OCR analysis from *Mfsd7cfl/fl* and *Mfsd7c*^-/-^ macrophages as measured by Seahorse XF96e Analyzer and their responses to oligomycin (oligo), FCCP, and rotenone plus antimycin A (Rot/AA), n=3 technical replicates (**a**), and their average basal and maximal OCR (**b**), n=3 biological replicates. (**c**) Representative ECAR analysis of *Mfsd7c^fl/fl^* and *Mfsd7c*^-/-^ macrophages and their responses to glucose, Rot/AA, and 2-deoxyglucose (2-DG). n=3 technical replicates. (**d**) Mitochondrial membrane potential of *Mfsd7c^fl/fl^* and *Mfsd7c*^-/-^ macrophages analyzed with TMRE staining followed by flow cytometry. (**e**) Cellular ATP/ADP ratio, n=3 biological replicates. (**f**) Estimates of cellular temperature of *Mfsd7c^+/+^* and *Mfsd7c^-/-^* macrophages as measured by fluorescent thermoprobe, n=3 biological replicates. *p<0.05, **p<0.01, ***p<0.005.

*7*

**Supplementary Fig. 6.** Construction of macrophage-specific *Mfsd7c* knockout mice, characterization and analysis of macrophages. (**a**) Schematic representation of wild-type (*Mfsd7c*^wt^), exon 2-floxed (*Mfsd7c*^fl^), and exon 2-deleted (*Mfsd7c*^-^) alleles. Primer set #1 and #2 and their respective PCR products used to distinguish between different alleles are shown. (**b**) PCR analysis of DNA from tails of wild-type (*Mfsd7c*^wt/wt^), floxed *(Mfsd7c^fl/fl^*) and heterozygous (*Mfsd7c^wt/fl^*) mice using primer set #1. (**c**) Schematic diagram showing construction of myeloid-specific Mfsd7c knockout mice. (**d**) Comparison of F4/80 and CD11b expression by *Mfsd7c^fl/fl^* and *Mfsd7c*^-/-^ bone marrow-derived macrophages (BMDM) by flow cytometry. (**e**) PCR analysis of genomic DNA isolated from tails and BMDM from floxed (*Mfsd7c*^fl/fl^) mice with and without LysM-Cre using primer set #2. (**f**) Quantitative RT-PCR analysis of *Mfsd7c* exons 1 and 2 with RNA isolated from *Mfsd7c^fl/fl^* and *Mfsd7c*^-/-^ BMDM. (**g**) Western blot analysis of *Mfsd7c^fl/fl^* and *Mfsd7c*^-/-^ BMDM lysates using antibodies against the C-terminal domain of MFSD7C and β-tubulin.

3. Moreover, is there evidence showing food intake would affect Heme levels in blood which in turn modulates thermogenesis?

**Response:**

There are two related questions here. The first one is whether food intake affects heme level in the blood. The second one is whether increased heme level in the blood modulates thermogenesis. Below, we address the two questions separately.

Red meats are most abundant in heme. Many epidemiological studies have examined the association of dietary heme intake, mainly from red meat, with risk of cancer, diabetes, and coronary heart diseases. But, to the best of our knowledge, none of them has actually measured heme levels in the blood and correlated the heme levels with the amounts of red meat consumption. Nevertheless, it is known that heme can be absorbed in the small intestines and that heme constitutes 95% of functional iron in the human body and two-thirds of the average person’s iron intake in the developed countries (Hooda et al 2014). Furthermore, once absorbed in the intestines, heme could be transported into the blood where it binds to hemopexin, albumin and other proteins in the serum, or transferred to other cells without going through the circulation. Under pathogenic conditions, such as sickle cell

8

anemia, the level of heme in the blood is elevated by two to ten-fold (Vinchi et al 2013; Keleku-Lukwete et al 2015).

Increased thermogenesis after a meal is an ancient phenomenon. In fact, food has been classified based on their thermogenic effects. Not surprisingly, meat, especially red meat, which is most abundant in heme, is most thermogenic. However, the molecular basis behind the increased thermogenesis after a meal is largely unknown. Suggestions include that i) heat production by the brown adipose tissue is activated after consumption of a meal, ii) metabolic rate is increased after a meal, and iii) heat from hot food is absorbed. Our finding suggests a new molecular mechanism of increased thermogenesis after meal: Heme from food is absorbed and can stimulate thermogenesis by uncoupling mitochondrial respiration. Our hypothesis is consistent with ancient observations that meat is the most thermogenic food.

In the revised manuscript, we have now discussed the implications of our findings on thermic effect of food more extensively.

Hooda J, Shah A, and Zhang L. Heme, an essential nutrient from dietary proteins, critically impacts diverse physiological and pathological processes. Nutrients. 2014, 6:1080-1102.

Vinchi F, De Franceschi L, Ghigo A, Townes T, Cimino J, Silengo L, Hirsch E, Altruda F, Tolosano E. Hemopexin therapy improves cardiovascular function by preventing heme-induced endothelial toxicity in mouse models of hemolytic diseases. Circulation. 2013; 127:1317-29.

Keleku-Lukwete N, Suzuki M, Otsuki A, Tsuchida K, Katayama S, Hayashi M, Naganuma E, Moriguchi T, Tanabe O, Engel JD, Imaizumi M, Yamamoto M. Amelioration of inflammation and tissue damage in sickle cell model mice by Nrf2 activation. Proc Natl Acad Sci U S A. 2015;112(39):12169-74.

**Reviewer #3 (Remarks to the Author):**

The manuscript by Li and colleagues reports on a new mechanism for thermogenesis, that can be regulated by heme binding to MFSD7C (aka FLVCR2). This is an interesting hypothesis and the in vitro data supporting heme binding to MFSD7C, and the interaction of MFSD7C to components of the ETC are in the most case strong. Likewise, the evidence that heme increases mitochondrial respiration and heat production is persuasive, although the use of the fluorescent polymeric thermometer requires further validation/explanation. The weakest part of the study concerns the mechanism by which MFSD7C increases thermogenesis and the (patho)physiological relevance of this pathway.

Major Points

1. The use of the fluorescent polymeric thermometer (FTP) reporter requires further explanation/validation. This would require more details of the synthesis of the reporter (details of the compound used). It is not clear to this reviewer how the fluorescent intensity can be used as a direct measure of temperature. Wouldn’t this require FLIM measurement (i.e. how can the uptake of the dye be assumed to be the same in the different cell lines)?

**Response:**

Fluorescence-lifetime imaging microscopy (FLIM) measures the lifetime of the fluorophore signal, rather than its intensity, and therefore is not affected by the difference in the uptake of fluorophore by different samples. We measured fluorescence intensity instead of lifetime due to a lack of access to FLIM. We showed that fluorescence is a direct measure of temperature by using cells incubated at different temperatures and generating a

9

temperature-standard curve (Supplementary Figure 4d), similar to ones reported in the literature (Okabe et al 2012). We also compared the fluorescent intensity of parental and 7CKO cells in the same culture dish to eliminate differences in experimental handling (Figure 2g and 4e). Furthermore, when we compared fluorescence intensity it is always between parental cell and 7CKO clones, which may minimize difference in uptake.

In addition to THP-1 cells and their KO clones, we used MCF7, 293T cells and the respective 7CKO clones. We also directly measured the temperature of culture media using thermal couples and observed consistent increase of temperature (0.15-0.3°C) in the 7CKO media relative to the parental THP-1 media.

To further address the reviewer’s concern, we have provided details of probe synthesis in the revised manuscript. In addition, we have used a commercial thermoprobe kit that contains an internal loading control fluorescence dye (Diagnocine, FDV-0005) in both cell lines and primary macrophages. These additional experiments show that 7CKO does not affect fluorescence probe uptake but consistently results in higher fluorescence intensity, i.e., temperature. These additional results are shown in Fig. 3 and Supplementary Fig. 7 (below) of the revised manuscript.

Together, these studies show that the use of fluorescence intensity is a reasonable measure of temperature.

**Supplementary Fig. 7.** (**a**) Schematic representation of the protocol used to analyze dye loading efficiency into BMDM and generation of the cellular temperature standard curve using the temperature-sensitive fluorescent properties of the thermoprobe dye. (**b** and **c**) Representative thermoprobe dye loading efficiency into *Mfsd7c*^fl/fl^ and *Mfsd7c*^-/-^ BMDM as analyzed by flow cytometry (**b**) and average of three biological replicates (**c**, error bars = standard deviation). (**d** and **e**) Comparison of thermoprobe fluorescence intensities in *Mfsd7c*^fl/fl^ (**d**) and *Mfsd7c*^-/-^ (**e**) BMDM at indicated incubation temperatures by flow cytometry. (**f**) Plot of incubation temperature versus mean fluorescence intensities (MFI) of thermoprobe loaded into *Mfsd7c*^fl/fl^ and *Mfsd7c*^-/-^ BMDM. Linear trendline (black dashed line) was used to convert MFI to relative cellular temperature of BMDC as shown in Fig. 3f.

Hayashi T, Fukuda N, Uchiyama S, Inada N. A cell-permeable fluorescent polymer for intracellular temperature mapping in mammalian cell lines. PLoS One. 2015; 10:e0117677.

10

Okabe K, Inada N, Gota C, Harada Y, Funatsu T, Uchiyama S. Intracellular temperature mapping with a fluorescent polymeric thermometer and fluorescence lifetime imaging microscopy. Nat Commun. 2012; 3:705.

2. The authors present extensive evidence for the interaction of MFSD7C with various proteins, but this is always determined using in vitro approaches. Can the authors provide evidence for these interactions in endogenous conditions (i.e. in mammalian tissues)?

**Response:**

Please see response to point #3 of Reviewer 1. As shown in Fig. 1f of the revised manuscript, we detected co-IP between endogenous MFSD7C with endogenous ATP5h, SERCA2b and Hmox1 in bone marrow-derived macrophages from normal mice, but not mice with *Mfsd7c* deletion in macrophages. Furthermore, the same as in cell lines with overexpression, the endogenous interactions are also sensitive to heme treatment. The interactions between MFSD7C and ATP5h and SERCA2b are disrupted by hematin treatment whereas interaction between MFSD7C and Hmox1 is enhanced by hematin treatment.

3. Related to the above point, the authors use primarily HEH293T and THP-1 cells, but what cell types/tissues do the authors imagine this mechanism might operate in? Do the authors have any evidence that the interaction is specific for SERCA2b? Other studies have reported that SERCA1a and SERCA2a are involved in futile calcium cycling and thermogenesis is skeletal muscle, whilst SERCA2b may be the predominant isoform responsible for calcium cycling in beige adipocytes. An important point in the current study would be to distinguish which isoforms are the most physiologically relevant (which in turn would probably reveal which tissues/cell types are important for this mechanism). In muscle, sarcolipin plays an important role in thermogenesis. Is there any evidence for a role of sarcolipin in the current study? Understanding these issues would require better characterisation of the endogenous levels of MFSD7C and components of the calcium cycling pathway (SERCA and the ryanodine receptors), rather than using in vitro cell models.

**Response:**

By IP-MS, we found that SERCA2b co-IP with MFSD7C. This interaction was further confirmed by co-IP by overexpressing MFSD7C and SERCA2b in cell lines as well as between endogenous MFSD7C and SERCA2b in primary macrophages. By IP-MS, we did not detect co-IP of SERCA1a and SERCA2a with MFSD7C, although SERCA1a and SERCA2a are expressed by THP-1 cells (see Figure below). Similarly, we do not have any evidence for the role of sarcolipin in our observations. We agree with the reviewer that further studies of MFSD7C with calcium cycling pathway (SERCA and the ryanodine receptors) in different tissues are critical to better understanding of the role of MFSD7C in thermogenesis and metabolism. However, we believe these studies are beyond the scope of the current study.

DMSO

Heme(10 uM)

Heme(40 uM)

1 1.1 1.4

1 2.4 5.4

1 1.2 1.5

Serca1

Figure: SERCA1a and SERCA2a protein levels in THP-1 cells. THP-1cells were either not treated or treated with the indicated concentrations of heme before lysis. Cell lysates were subjected to Western blotting with anti-SERCA1 (CST, 12293), anti-SERCA2 (ab2861) and anti-®-tubulin. Shown are representative data from one of two experiments.

Serca2 α-tubulin

11

4. Why is the ATP to ADP ratio so low (Fig. S4b)? ATP levels would be expected to be 5-10 fold higher than ADP levels.

We thank the reviewer for raising this issue. After testing several kits from different manufacturers we decided to include data generated with Millipore Sigma ATP/ADP Ratio Assay Kit (#MAK135). Our new data indicates that our cell clones contain ~200 pmol ATP per 100,000 cells which would be equal to a ~ 2 mM ATP concentration per cell. Parental THP-1 cells have ATP/ADP ratio of ~4, while all 7CKO clones have significantly lower ATP/ADP ratio of ~3.5 (Figure 2f) (see below). As another independent analysis, we performed targeted metabolomic of parental THP-1 cells and one of the 7CKO clones (B11) to measure the relative amounts of ATP, ADP, and AMP (Supplementary Fig. 4b and 4c) (See below). Consistent with the rest of our dataset, parental THP-1 cells had ATP/ADP ratio of ~4, while 7CKO clone had lower ATP/ADP ratio, but higher ATP/AMP ratio suggesting that 7CKO cells indeed have compromised ATP synthesis and/or increased ATP consumption.

**Fig. 2f.** Cellular ATP/ADP ratio, average and standard deviation from five technical repeats are shown.

**Supplementary Fig. 4b,c**. Targeted metabolomic analysis of ATP, ADP, and AMP levels (b) and ATP/ADP ratio of parental THP-1 and 7CKO clone B11 cells (c) (100,000 cells each, n=3 independent experiments).

**Reviewer comments, second version:**

Reviewer #1 (Remarks to the Author):

I have to apologize for my error (“reduced cell respiration” instead of “reduced ATP synthesis”) in the previous review.
The authors addressed all my previous criticisms. They demonstrated that MFSD7C resides in mitochondria and that the endogenous protein co-immunoprecipitate with mitochondrial proteins, SERCA2b and Hmox1.
Data are clearly presented and well discussed.


Reviewer #2 (Remarks to the Author):

Comments are adequately addressed.


Reviewer #3 (Remarks to the Author):

The revised version of the manuscript addresses all of my comments. I would just raise one minor, but I think important, point that relates to the issue of the isoforms/cell types involved in this new thermogenic regulatory pathway. In the introduction, the authors state that the heater organs of some species of fish utilise SERCA2b (and use this argument to support the current study). However, I do not agree with this interpretation (and I don’t think the references that are cited support this claim either). My understanding is that the heater organs utilise Serca 1. In this regard, it would be relevant for the authors to briefly mention in their discussion a recent study in mice showing that Serca1 appears to be involved in a Ucp1-independent thermogenic mechanism in white adipose tissue (Pollard et al. Nature Metabolism, 1, 340-349).

**Author rebuttal, second version:**

Reviewer #1 (Remarks to the Author):

I have to apologize for my error (“reduced cell respiration” instead of “reduced ATP synthesis”) in the previous review.

The authors addressed all my previous criticisms. They demonstrated that MFSD7C resides in mitochondria and that the endogenous protein co-immunoprecipitate with mitochondrial pro­teins, SERCA2b and Hmox1.

Data are clearly presented and well discussed.

Response: We thank the reviewer for helpful critique of our work.

Reviewer #2 (Remarks to the Author):

Comments are adequately addressed.

Response: We thank the reviewer for helpful critique of our work.

Reviewer #3 (Remarks to the Author):

The revised version of the manuscript addresses all of my comments. I would just raise one mi­nor, but I think important, point that relates to the issue of the isoforms/cell types involved in this new thermogenic regulatory pathway. In the introduction, the authors state that the heater or­gans of some species of fish utilise SERCA2b (and use this argument to support the current study). However, I do not agree with this interpretation (and I don’t think the references that are cited support this claim either). My understanding is that the heater organs utilise Serca 1. In this regard, it would be relevant for the authors to briefly mention in their discussion a recent study in mice showing that Serca1 appears to be involved in a Ucp1-independent thermogenic mechanism in white adipose tissue (Pollard et al. Nature Metabolism, 1, 340-349).

Response: We have corrected the error by stating that SERCA1 isoform is involved in thermo-genesis in fish heater organs, and included a potential role of Serca1 in thermogenesis of white adipocytes in the Introduction as follows: “Recently, SERCA2b was shown to be required for thermogenesis in beige adipocytes of *Ucp1^-/-^* mice and in pigs^10^, which lack a functional copy of *Ucp1*, while SERCA1 may stimulate thermogenic activity in white adipocytes in mice11.”

**Reviewer comments, third version:**

**Author rebuttal, third version:**
